# An Update of Fungal Endophyte Diversity and Strategies for Augmenting Therapeutic Potential of their Potent Metabolites: Recent Advancement

**DOI:** 10.1007/s12010-024-05098-9

**Published:** 2025-02-05

**Authors:** Chandrabhan Prajapati, Sachchida Nand Rai, Anurag Kumar Singh, Balu A. Chopade, Yashveer Singh, Santosh Kumar Singh, Shafiul Haque, Miguel Angel Prieto, Ghulam Md Ashraf

**Affiliations:** 1 Centre of Experimental Medicine and Surgery, Institute of Medical Sciences, Banaras Hindu University, Varanasi, 221005 India; 2 Department of Pharmaceutical Engineering & Technology, Indian Institute of Technology (BHU), Varanasi, 221005 India; 3 AKS University, Satna, Madhya Pradesh 485001 India; 4 Department of Statistics, Institute of Science, Banaras Hindu University, Varanasi, 221005 India; 5 Research and Scientific Studies Unit, College of Nursing and Allied Health Sciences, Jazan University, 45142 Jazan, Saudi Arabia; 6 Nutrition and Bromatology Group, Analytical and Food Chemistry Department. Faculty of Food Science and Technology, University of Vigo, Ourense Campus, E-32004 Ourense, Spain; 7 Department of Biosciences and Bioinformatics, School of Science, Xi’an Jiaotong-Liverpool University, 111 Ren’ai road, SIP, Jiangsu Province, Suzhou, 215123 P. R. China

**Keywords:** Fungal endophytes, OSMAC, Co-culture, Biosynthetic gene clusters (BGCs), Bioactive compounds

## Abstract

Endophytic fungi represent a significant renewable resource for the discovery of pharmaceutically important compounds, offering substantial potential for new drug development. Their ability to address the growing issue of drug resistance has drawn attention from researchers seeking novel, nature-derived lead molecules that can be produced on a large scale to meet global demand. Recent advancements in genomics, metabolomics, bioinformatics, and improved cultivation techniques have significantly aided the identification and characterization of fungal endophytes and their metabolites. Current estimates suggest there are approximately 1.20 million fungal endophytes globally, yet only around 16% (190,000) have been identified and studied in detail. This underscores the vast untapped potential of fungal endophytes in pharmaceutical research. Research has increasingly focused on the transformation of bioactive compounds by fungal endophytes through chemical and enzymatic processes. A notable example is the anthraquinone derivative 6-O-methylalaternin, whose cytotoxic potential is enhanced by the addition of a hydroxyl group, sharing structural similarities with its parent compound macrosporin. These structure-bioactivity studies open up new avenues for developing safer and more effective therapeutic agents by synthesizing targeted derivatives. Despite the immense promise, challenges remain, particularly in the large-scale cultivation of fungal endophytes and in understanding the complexities of their biosynthetic pathways. Additionally, the genetic manipulation of endophytes for optimized metabolite production is still in its infancy. Future research should aim to overcome these limitations by focusing on more efficient cultivation methods and deeper exploration of fungal endophytes’ genetic and metabolic capabilities to fully harness their therapeutic potential.

**Graphical Abstract:**

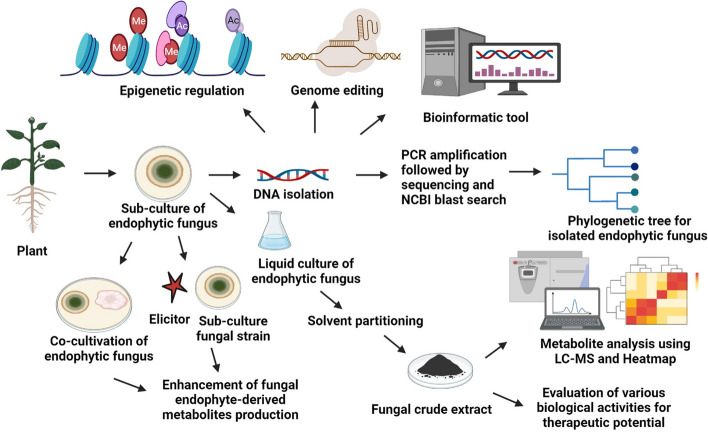

## Introduction

**Fig. 1:**
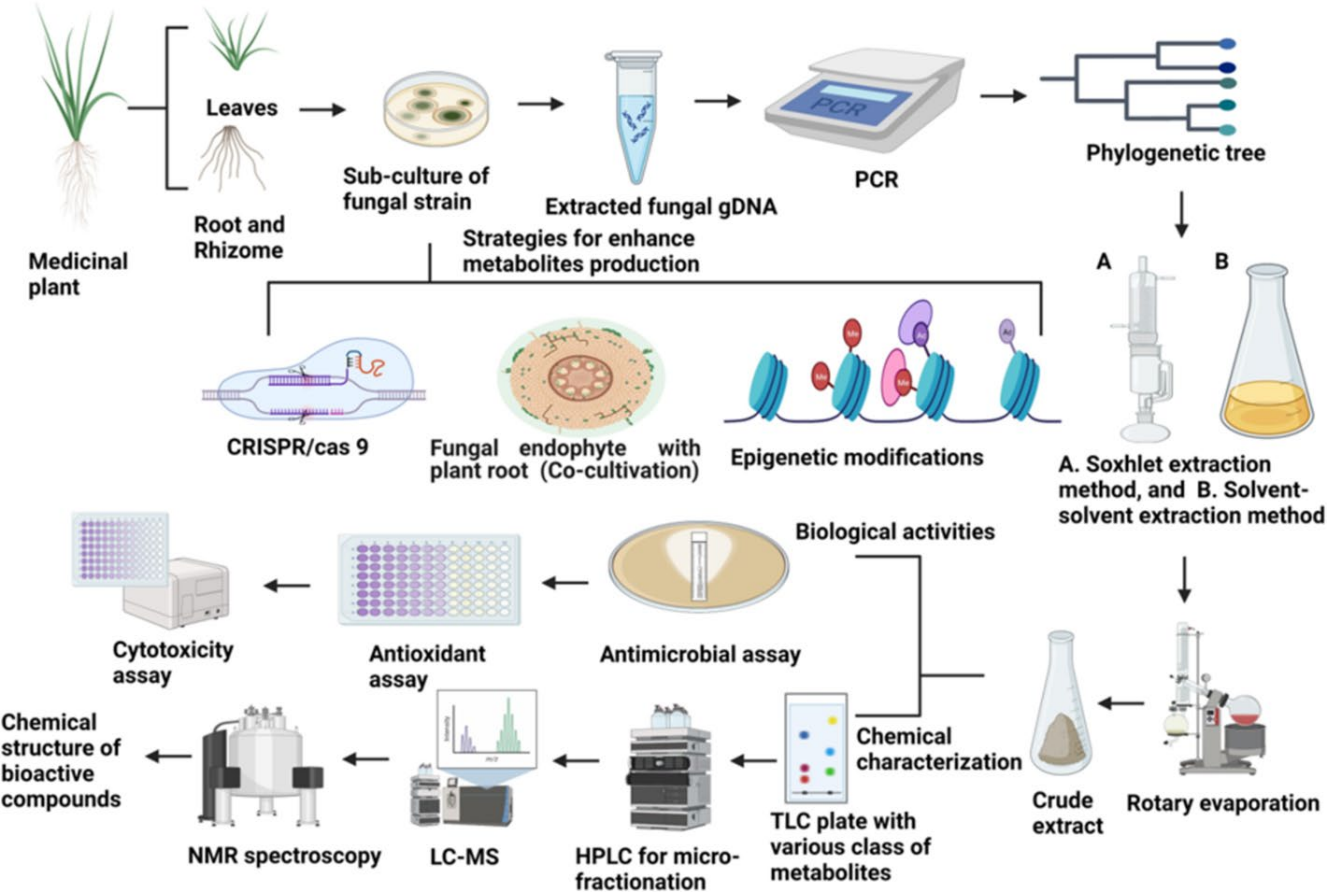
Elucidates the isolation as well as characterization of endophytic fungal-derived potent bioactive metabolites and various approaches involved to improve the biosynthesis of metabolites. This figure represents the processes involved in the isolation, identification, and mass production of fungal endophyte-derived compounds with various bioactivities including antimicrobial, antioxidant, cytotoxicity, etc. Take the sterilized parts of the targeted plant and cut them into small pieces; further place these small plant pieces onto potato dextrose agar (PDA) plates supplemented with antibiotics. Allow fungal mycelia to emerge at ambient temperature and then transfer the emerged fungal colonies onto fresh PDA plates to obtain single fungal strains. The fungus is identified by their morphological and molecular characteristics. There are two methods for metabolite extraction, namely, Soxhlet extraction and solvent–solvent extraction that is utilized as per requirements. Mostly fungal metabolites are extracted using the solvent–solvent extraction method from broth cultures of fungi. The crude extract may contain some solvent residues which are removed using vacuum rotary evaporation. The dried crude metabolites are then analyzed on TLC plates to identify various classes of metabolites by using the specific detecting reagents. Fractionation of the crude extract using HPLC and further assessed their potential for biological activities through bioactivity-guided fractionation. The purified bioactive compounds are subsequently chemically characterized through spectroscopic analyses like mass spectrometry (MS) along with nuclear magnetic resonance (NMR) spectroscopy. Co-cultivation of fungus with roots of plants offers advantages because it promotes plant growth, facilitates nutrient absorption, and strengthens disease resistance. Fungi, specifically mycorrhizal forms, create symbiotic associations with plant roots, allowing plants to uptake vital nutrients such as phosphorus whereas getting sugars in return. This relationship also enhances soil fertility along with structure while boosting the plant’s resistance to environmental challenges. Co-cultivation can also result in the development of valuable secondary metabolites, which can help in bioremediation actions by degrading soil pollutants. Various approaches may be utilized for increased production of potent metabolite linked with fungal entophytes using the genome-editing CRISPR/Cas9 technique; co-cultivation with other fungi, bacteria, or plants; and chromatin remodeling, etc

Nature has a treasure of tremendous resources for research and development based on plant and their products. Natural resources used in traditional therapies are now utilized for the innovation and development of modern medication [1, 2]. Health issues can also depend on the diet intake [3]. Developing drug resistance remains a big challenge to drug developers to date [4]. A number of plants, animals, or microbes may produce pharmaceutically important bioactive compounds through their primary and secondary metabolism. The total number of plant-based compounds had reached one million because of technological advancements in screening programs, and also in extraction and separation processes [5, 6]. Plant-associated metabolites were discovered by studying 5 to 15% of higher plants and approximately 5 to 7% of microbe-derived compounds. Naturally derived chemicals which are less studied offer promising opportunities for research and development of novel bioactive molecules [7, 8]. Endophytes constitute a polyphyletic group comprising various microbes that dwell within the healthy tissue of plants and produce favorable impacts on their plant host. They develop a variety of complex biological intra and inter-associations with the plant host [9–11]. Endophytes have capabilities to producing a distinct spectrum of bioactive chemicals having a number of biological characteristics [12–15]. Endophytic fungi found in medicinal plants possess the potential to generate bioactive chemicals having structurally and chemically identical to compounds produced by their host plant [16]. The landmark discovery in 1993 revealed the production of *Taxus brevifolia* plant-associated anticancer chemical “Taxol” through their endophytic fungus *Taxomyces andreanae* [17]. This discovery sparked an interest in fungal endophytes for isolating and characterizing the plant-derived therapeutic molecules [18–21]. The potential that enables a fungal endophyte to generate bioactive chemicals comparable to that of its host species could be linked to a process of horizontal transfer of genes [22]. The asexual form of fungal endophytes of grasses may also interact via vertical gene transfer processes [23]. Researchers are using endophytic fungi as a viable resource for natural bioactive compounds to meet the opportunity appearing to broad interest [24–28]. Taxol has now become a billion-dollar anticancer medicine in the world to treat cases of pulmonary, breast, and ovarian malignancies, as well as polycystic kidney disease, which are widely utilized in healthcare facilities [29]. Taxol may also be produced by another fungal endophyte, like *Epicoccum nigrum* TXB502, a fungal endophyte dwelling within the *Taxus baccata* plant that produces the anticancer chemical Taxol [30]. Another anticancer chemical, *N*-(2-hydroxyethyl) hexadecanamide (palmitoylethanolamide, PEA), had recently been discovered in the leaf-associated endophytic fungal strain *Colletotrichum gloeosporioides* that survives in the host plant *Oroxylum indicum* [31]. Fungal endophyte-derived compounds have distinct biological activities comprising antioxidants, antimicrobial, cytotoxic, anticancerous, antidiabetic, neuroprotective, and antihypercholesterolemic etc., [32–38] (Fig. 1). We have explored the information on fungal endophyte-derived compounds having bioactivities such as antioxidant, antibacterial, cytotoxic, anticancer, antidiabetic, neuroprotective, and antihypercholesterolemic properties (Figure. 3 and Figure. 4). We have discussed here the biological activity of fungal endophyte-derived metabolites in vitro and in vivo conditions. We have also discussed several potent bioactive compounds produced from fungal endophytes and their mechanisms of action along with signaling pathways. The OSMAC method, which refers to “one strain many compounds,” has been used in the discovery of microbial natural products. It means culturing a single microbe under a variety of environmental and nutritional conditions in order to trigger the synthesis of numerous secondary bioactive metabolites [39]. The concept indicates that altering parameters such as nutrition supplies, pH level, temperature, or co-culture partners may stimulate the microbe to create a wide range of compounds because it would not generally produce under ordinary laboratory settings [40]. This technique becomes useful for identifying novel bioactive compounds from identified microbes, including antimicrobial agents, anticancer compounds, and other pharmaceutically important products [41, 42]. Researchers have employed OSMAC, co-cultivation, epigenetic modifiers, pleiotropic regulators, elicitors, and molecular techniques involved in enhancing secondary metabolite synthesis by activating silent biosynthetic gene clusters (BGCs) in fungi. This manuscript focuses on investigating the therapeutic potential of compounds derived from endophytic fungi, aiming to develop effective and advanced treatments for diseases such as microbial infections, cancer, diabetes, and neurodegenerative conditions. The primary objectives include identifying bioactive compounds with antimicrobial, antioxidant, antidiabetic, anticancer, antimalarial, neuroprotective, and cholesterol-lowering properties. The study utilizes a combination of bioactivity screening, metabolite profiling, and molecular pathway analysis to understand how these fungal endophyte-derived compounds exert their effects. By studying how these compounds work, the research aims to help in discovering new drugs and tackling issues such as drug resistance and the lack of effective treatments for certain diseases.

## Fungal Endophyte Bioprospecting: Upstream and Downstream Processes, Yield, Yield Coefficient, Conditions for Cultivation

Upstream processing regarding fungal endophyte bioprospecting comprises the first stages of isolating and characterizing endophytic fungi from the plant samples, then optimizing the cultivation parameters [43]. At this step, the fungi’s development within bioreactors can also be accelerated in order to generate more bioactive chemicals. The aim seems to create the optimal environment for fungal growth along with metabolite synthesis, which includes the appropriate medium, pH level, and temperature conditions as well as levels of oxygen [44–46].

Following the production of the bioactive chemicals, downstream processing includes removing the biomass of fungi from the growth medium, extracting the essential chemicals, and purification of chemicals as well [47]. Methods like solvent extraction as well as chromatography are utilized frequently [48, 49]. The last steps can include transforming the purified components into a useful product form, assuring their high quality along with suitability for future use [50].

The yield value refers to the total amount of metabolites acquired via the fungal culture, whereas the yield coefficient seems to be the fungal efficiency with which substrate can be transformed into the product of choice in cultured fungal biomass. These indicators are essential for evaluating the bioprospecting process’s feasibility along with efficiency [51]. The amount of a particular bioactive substance that the fungal endophyte produces during culture can be referred to as the yield concerning fungal endophyte bioactive metabolites. It is commonly represented as the quantity of metabolite acquired per unit mass of endophytic fungus biomass as well as per unit volume concerning medium for culture, represented in grams or milligrams [52]. In the scenario of endophytic fungal metabolites, the yield coefficient measures the connection between the quantity of metabolite synthesized in comparison with the total amount of substrates utilized, including nutrients as well as carbon sources [53]. It is often represented by a ratio, for example, Y_P/S, in which Y_P/S defines the quantity of product (metabolite) produced per unit of substrates utilized. In order to maximize production and comprehend the effectiveness of metabolite synthesis via the endophytic fungi, it is essential to know the yield coefficient [54, 55].

The term “culture conditions” refers to the combination of environmental along with nutritional parameters, comprising the temperature level of the incubator, pH level of the culture medium, optimum levels of oxygen during cultivation, along with incubation time, affecting fungal growth along with metabolite synthesis [56, 57]. Ensuring the effective production of crucial bioactive chemicals from fungal endophytes as well as improving yield depends on optimizing these circumstances [43]. Thus, fungal endophyte bioprospecting, optimizing upstream as well as downstream processes, maximizing yield, along with modifying culture conditions are all essential for appropriately discovering and synthesizing bioactive chemicals.

The authors present an in-depth analysis of fungal endophyte bioprospecting. They effectively underscore the importance of fine-tuning both upstream and downstream processes to maximize the efficiency of bioactive chemical production. This includes optimizing culture conditions—such as temperature, pH, and oxygen levels—as well as refining the methods for biomass removal, metabolite extraction, and purification. They also emphasize the significance of yield metrics, which gauge both the quantity of metabolites produced and the efficiency of substrate conversion. Such a detailed approach is crucial for advancing the discovery and synthesis of valuable bioactive compounds from fungal endophytes.

## Molecular Mechanisms Underlying Fungal Endophyte-Derived Metabolite Production

Fungal endophytes use a number of molecular processes that control how they synthesize and release bioactive substances to produce metabolites via fermentation. These intricate processes involve the coordination of regulatory, enzymatic, and genetic processes inside the fungal cells [58]. An outline of the fundamental molecular processes is described below:

### Regulation and Expression of Genes

Fungal endophytes have complex genetic regulation controlling their metabolite synthesis. Certain genes produce the enzymes that participate in the processes of biosynthesis leading to the creation of metabolites [59]. Various environmental conditions, including temperature, pH, oxygen supply, and nutrition availability, regulate whether such gene clusters express themselves in fungi [60–62]. In response to certain external stimuli, regulatory proteins—such as transcription factors—play a critical role in activating or deactivating fungal gene clusters [63]. Global regulators as well as pathway-specific regulators can frequently coordinate and control the activation of deactivation gene clusters for secondary metabolite, known as collections of genes that produce a particular metabolite[58].

### Biosynthesis Pathways

Simple substrates (such as sugars and amino acids) are transformed into complex metabolites via a sequence of biochemical processes that are catalyzed by the enzymes encoded by the corresponding genes whenever the substances are gathered [64]. These biosynthetic routes frequently include several stages which are typically catalyzed by a particular enzyme and therefore are very specific. For instance, non-ribosomal peptide synthetases (NRPSs) as well as polyketide synthases (PKSs) are essential enzymes in the synthesis of several beneficial secondary metabolites. In order to create the product, these kinds of enzymes improve along with systematically transforming molecular building components[65].

### Enzymatic Alterations

In addition to assembling the metabolites, enzymes also carry out modifications on them, including acylation, glycosylation, hydroxylation, and methylation. The final metabolite’s biological activity and stability, along with solubility, depend on these alterations. Enzymes accountable for these alterations are frequently encoded under specific gene clusters as the fundamental biosynthetic enzymes, ensuring a strong correlation between the changes and the synthesis of metabolites [66].

### Control in Transport and Secretion Level

Metabolites can get transmitted from the fungal cells to surrounding media following their synthesis. Particular transport proteins can actively transfer the metabolites through the cell membrane, involving ABC (ATP-binding cassette) transporters, and can frequently involve in this process. Secretion is needed not only for metabolite collection throughout the culture medium but also for avoiding metabolite-induced toxicity in the generating fungal cells [67].

### Signal Transduction Processes

Metabolite synthesis can also be controlled via signal transduction processes, which act as internal communication networks in fungal cells and depend on surroundings factors. Receptor proteins that sense environmental changes, including nutritional depletion or stressful situations, and transmit this information via a series of signaling molecules are frequently involved in such pathways. This eventually can result in either the activation or suppression of genes that regulate metabolite synthesis [68, 69].

### Epigenetic Modifications

The expression of genes involved in the biosynthesis of metabolites can sometimes be regulated by epigenetic processes such as DNA methylation as well as histone remodeling. Without changing the basic genetic coding, these changes can affect gene expression by changing the DNA’s accessibility towards transcription machinery. Fungal endophytes use epigenetic control to dynamically control metabolite synthesis in response to changing environmental circumstances [70].

### Interaction Between the Environment and the Host

Interactions between fungal endophytes together with the plants they inhabit, as well as the environment around them, have a significant impact on the development of metabolites via fermentation [71]. Hormones as well as root exudates are examples of host-plant interactions that can stimulate or decrease the synthesis of certain metabolites via activation or suppression of relevant genes of endophytic fungi [72, 73]. Environment-related factors like as pH, temperature, along with nutrient availability, are also very important since they frequently cause stress-related responses which lead to the creation of bioactive chemicals [74]. Furthermore, interactions—whether cooperative or competitive—with other microbes can change the process by which metabolites are produced, possibly leading to the emergence of new compounds [75]. Such dynamic interactions are important factors regarding fungal endophyte bioprospecting due to their optimized metabolite production along with diversity.

To summarize, the complex molecular mechanisms that include gene expression, enzyme activity, regulation pathways, along with environmental interactions, lead to the synthesis of metabolites produced from fungal endophytes during fermentation. Gaining insight into these mechanisms might be essential to improving fermentation processes in addition to increasing the diversity and yields of bioactive metabolites.

The authors thoroughly examine the complex molecular mechanisms of fungal endophytes in metabolite synthesis. They highlight the roles of gene regulation, biosynthesis pathways, enzymatic modifications, transport, signal transduction, and epigenetic changes. Environmental and host interactions also significantly influence metabolite production, emphasizing the need for deeper insights to optimize fermentation processes and enhance bioactive metabolite yields.

## Estimated Population of Fungal Endophytes on Earth

**Fig. 2:**
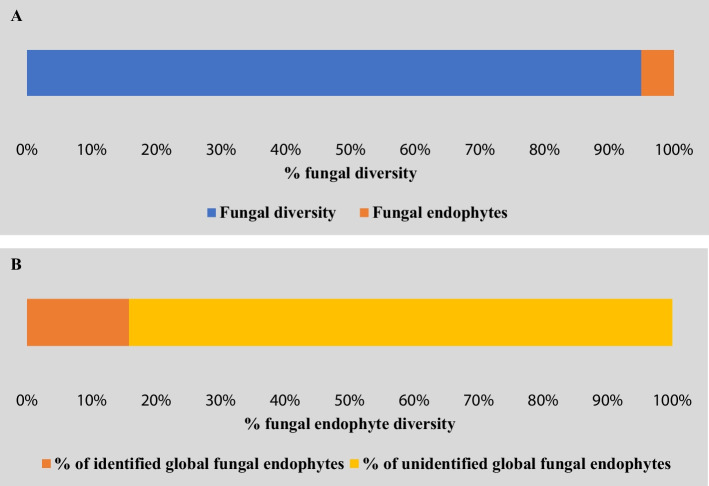
**A** Percent of the identified fungal endophytes among total fungal diversity as per suggested data of Hawksworth and Lücking in 2008. **B** Percent of identified and unidentified fungal endophytes estimated using the 95% confidence interval statistical method based on secondary data collected from 50 medicinal plants across various global geographical regions

In nature, almost every plant has been observed to be harboring a minimum of one symbiotic fungal endophyte [76]. However, the colonization and richness of the endophytic fungus depends on spatial location and environmental factors around the host plant [77–79]. The plant’s genetic makeup is crucial for how fungal endophytes colonize and interact with it. Thus, genotypic factors play a key role in host-endophyte interaction [80–82]. Endophytic fungus colonization within their plant host can be attributed to various factors such as canopy cover, plant age, different plant parts, leaf structure, and variations in climatic conditions [83–85]. Hawksworth and Lücking proposed that fungi, estimated to range from 2.2 to 3.8 million species, represent the largest number among all organisms globally. They also suggested in 2008 that fungal endophytes make up around 5% of fungal diversity [86, 87]. Ongoing research on fungal endophytes suggests that a significant number of these organisms remain unidentified to date [88]. According to the Botanical Survey of India, the number of identified plants in India is approximately 44,500, accounting for 7% of the world’s plant species (https://bsi.gov.in/page/en/national-wild-life-action-plan). We used secondary data from 50 medicinal plants across different geographical areas worldwide to estimate the total number of fungal endophytes expected to inhabit plants globally. Using the 95% confidence interval statistical method for estimation, we found that each plant contains a minimum of 2, an average of 30, and a maximum of 58 fungal endophytes. If we consider approximately 3.8 million fungal species, around 5% (Fig. 2A), which may be 0.19 million, could represent the identified fungal endophytes. We also found that minimum of ~ 1.20 million fungal endophytes might present globally. Resulting in about 1.01 million, i.e., about 84% of total expected fungal endophyte diversity might be unidentified to date (Fig. 2B). This study might offer researchers a great opportunity to identify and characterize fungal endophytes.

The authors highlight that nearly every plant harbors at least one fungal endophyte, with colonization influenced by genetic and environmental factors. They estimate that, globally, approximately 1.2 million fungal endophytes exist, with about 84% remaining unidentified. This underscores the vast potential for future research and discovery in fungal endophyte diversity.

## Fungal Endophyte-Derived Compounds with Antimicrobial Activities

A natural or chemically synthesized compound that destroys or hinders the growth and development of microorganisms including bacteria and fungi is known as an antimicrobial compound. Various antimicrobial chemicals have been categorized based on their distinct functions or mode of action. One category encompasses agents that hinder the formation of bacterial cell walls, including vancomycin, beta-lactams, and fosfomycin. Another group involves substances that impede protein production, such as tetracyclines, macrolides, and aminoglycosides. Additionally, there are compounds like 4-quinolones that inhibit DNA synthesis and others like rifampicin that halt RNA synthesis [89]. Numerous antibacterial compounds have exhibited antibacterial effects towards *Bacillus subtilis* through some modes of action, particularly cell wall biosynthesis inhibition, class II topoisomerases, protein, fatty acid, and folic acid biosynthesis, along with ionophores and detergents [90]. Now, fungus mRNA splicing along with bacterial DNA synthesis is being used as a target site for antimicrobial compounds [91–94]. The riboswitching approach is a new area for antimicrobial drug development [95]. The fungal endophyte *Phoma* sp., residing within the leaf tissues of the *Glycyrrhiza glabra* Linn. plant, generated two thiodiketopiperazine derivative compounds known for their antimicrobial effects. Both compounds exhibited antibacterial activities against pathogenic bacteria, particularly *Staphylococcus aureus*, as well as *Streptococcus pyogenes*, having an IC_50_ value under 10 μM. The in vitro study revealed that both compounds had bactericidal activity, bacterial transcription/translation inhibition activity, and inhibition of staphyloxanthin synthesis in *Staphylococcus aureus* [96]. The fungal endophyte *Penicillium janthinellum* SYPF 7899, found within the host plant *Panax notoginseng*, generated the bioactive compound Brasiliamide J which exhibited significant antibacterial effect towards *Bacillus subtilis* along with *Staphylococcus aureus* with MIC values of 15 and 18 μg/ml, respectively, and also alter the morphology of both bacteria. The computational findings indicate that both Brasiliamide J along with its rotamer demonstrate significant binding energies, forming strong hydrogen bonds as well as hydrophobic interactions at the binding sites within the protein FtsZ (filamenting temperature-sensitive protein Z) [97]. A fungal endophyte *Penicillium setosum* had potency to produce secondary metabolites with antimicrobial action. The crude extract of *Penicillium setosum* showed considerable antibacterial activity towards *Escherichia coli* as well as *Staphylococcus aureus.* The in vitro and in silico study revealed that *Penicillium setosum* show antimicrobial action via bacterial morphological alteration, Na^+^ and K^+^ ion regulation through bacterial membrane, and upregulation of β-galactosidase production. The active fraction of crude extract of *Penicillium setosum* was obtained through HPLC microfractionation. The active fraction displayed notable antibacterial effect towards *Escherichia coli* and *Staphylococcus aureus* with a MIC (minimum inhibitory concentration) value of 8 μg/mL. The LC-HR-MS data revealed that the active fraction contains plant metabolites, namely, leucodelphinidin and dihydroquercetin, along with kaempferol, quercetin (22) (Fig. 4), and patulin. The in silico study revealed leucodelphinidin, and dihydroquercetin had significant binding interaction with several antimicrobial targets of FabG (1I01), FabZ (1U1Z), FabI (5CG1), D-ALA:D-ALA ligase (2I80), penicillin-binding protein (1VQQ), DNA gyrase (2XCS), dihydropteroate (1AJO), and 16S rRNA A site (1MWL) bonded along with EFTu (1OB2)[98]. An endophytic fungus *Phomopsis* sp. that dwells inside the plant host *Salix gracilistyla* var. *melanostachys* was found to produce an antimicrobial compound phomopsichalasin which on structural characterization represented a novel cytochalasin-type compound, but unlike typical cytochalasins, it possesses a unique three-ring system in place of the traditional cytochalasin macrolide ring. This compound exhibited a considerable antimicrobial effect towards bacterial species comprising *Bacillus subtilis*, *Staphylococcus aureus*, and *Salmonella galinarum*, along with the fungal species *Candida tropicalis* [99]. An endophytic fungal strain *Aspergillus versicolor* Eich.5.2.2., isolated from the flower tissue of the *Eichhornia crassipes* plant produced a new compound, 22S-aniduquinolone A, along with an isomer Aniduquinolone A. Both compounds exhibited potent antibacterial effect towards *Staphylococcus aureus* (ATCC700699) having a MIC value of 0.4 µg/mL. This study suggested that both compounds may work synergistically to show antibacterial activity [100]. An endophytic fungus *Acremonium* sp., which survives in the host plant *Taxus baccata* generated a few antifungal and anticancer peptides known as leucinostatins. Leucinostatin A particularly exhibited significant antifungal activity against another fungus *Pythium ultimum*. NMR along with MS spectroscopic method was used to determine the molecular structure of the compound leucinostatin A[101]. Lu et al. demonstrated *Colletotrichum* sp., a fungal endophyte, dwells in the *Artemisia annua* plant. This endophytic fungus produces several bioactive compounds, namely, ergosterol (1), 3β,5α,6β-trihydroxyergosta-7,22-diene (2), 3β-hydroxy-ergosta-5-ene (3), 3-oxo-ergosta-4,6,8(14),22-tetraene (4), 3β-hydroxy-5α,8α-epidioxy-ergosta-6,22-diene (5), 3β-hydroxy-5α,8α-epidioxy-ergosta-6,9(11),22-triene (6), 3-oxo-ergosta-4-ene (7), 6-isoprenylindole-3-carboxylic acid (8), 3β,5α-dihydroxy-6β-acetoxy-ergosta-7,22-diene (9), 3β,5α-dihydroxy-6β-phenylacetyloxy-ergosta-7,22-diene (10), and a phytohormone indole-3-acetic acid (IAA). The structure of new bioactive compounds was identified by the combination of spectroscopic methods such as ^1^H NMR, ^13^C NMR, and IR, along with MS. The compounds 3–5 and 8–10 exhibited antibacterial effects towards *Bacillus subtilis*, *Staphylococcus aureus*, and *Sarcina lutea*, along with *Pseudomonas* sp. exhibiting MIC values ranging between 25 and 75 μg/ml. However, compounds 3, 5, 9, and 10 discussed previously exhibited antifungal effects towards *Candida albicans* and *Aspergillus niger* exhibiting MIC values ranging between 50 and 100 μg/ml. Compounds 3, 4, 8, 9, and 10 exhibited fungistatic effects towards the other crop pathogenic fungi *Gaeumannomyces graminis* var. *tritici*, *Rhizoctonia cerealis*, and *Helminthosporium sativum*, as well as *Phytophthora capisici* [27]. The fungal endophyte *Gliocladium* sp., isolated from the plant *Eucryphia cordifolia*, produces many volatile organic compounds (VOCs) with antimicrobial properties. The compound 1,3,5,7-cyclooctatetraene, known as Annulene derived from *Gliocladium sp.*, exhibited a selective antimicrobial effect against the fungus *Pythium ultimum* and *Verticillium dahlia* [102]. The fungal endophyte *Chalara* sp., dwelling in the *Artemisia vulgaris* plant, produces four bioactive compounds such as isofusidienol A, isofusidienol B, isofusidienol C, and isofusidienol D. The isofusidienols have antifungal effects towards another fungus *Candida albicans* along with antibacterial activity towards gram-positive and gram-negative bacteria [103]. The fungal endophyte *Trichoderma erinaceum* had been obtained from healthy bean field crops and produced a new compound (Z)−5-amino-5-(1,1,2-trihydroxybuta-1,3-dienyloxy)pentane-6,7,8,9-tetraol and five previously identified compounds. Ethyl acetate extract along with purified compounds obtained from *Trichoderma erinaceum* exhibited significant antifungal activity towards *Pythium ultimum* [104]. Another recent study showed that six endophytic fungi, namely, *Fusarium solani*, *Talaromyces trachyspermus*, *Aspergillus cejpii*, *Talaromyces assiutensis*, *Saccharomycopsis fibuligera*, and *Aspergillus niger*, were isolated from the tissues of the root, stem, and leaf of the host plant *Hedera helix*. Among isolated endophytes, the ethyl acetate extract of *Aspergillus cejpii* exhibited potent antimicrobial activity against *Escherichia coli*, *Pseudomonas aeruginosa*, *Staphylococcus aureus*, *Serratia marcescens*, *Acinetobacter baumannii*, and *Salmonella typhi* with MIC values ranging from 62.5 to 125 µg/ml. Spiculisporic acid, a potent bioactive compound, was isolated from the active fraction of *Aspergillus cejpii* via a bioactive-guided fractionation. Its structure was elucidated using spectroscopic techniques such as ^1^H and ^13^C-NMR analysis. Spiculisporic acid exhibited significant antimicrobial activity against pathogenic strains such as *E. coli*, *P. aeruginosa*, *S. aureus*, *S. marcescens*, *A. baumannii*, and *S. typhi*, along with multidrug resistant (MDR) strains comprising methicillin-resistant *S*. *aureus* (MRSA, H1), *P. aeruginosa* (PS 16), and *A. baumannii* (ACT 322) with MIC values ranging from 3.80 to 31.133 µg/ml [105]. Endophytic fungus derived several potent bioactive compounds with various classes of compounds including aliphatic, alkaloids, peptides, and phenolics; polyketides along with terpenoids have antimicrobial activities, but their mode of action was less explored [106–114]. Thus, antibacterial effects may be due to bacterial cell wall biosynthesis inhibition, DNA synthesis inhibition, transcription, translation process inhibition, and several proteins inhibition. However, antifungal effects may be due to disrupted fungal mRNA splicing and sporulation inhibitions. The authors review diverse antimicrobial compounds from fungal endophytes, noting their varied mechanisms of action including cell wall biosynthesis inhibition, DNA and RNA synthesis disruption, and protein inhibition. They highlight significant findings such as novel compounds with potent antibacterial and antifungal activities, emphasizing the need for further exploration of their modes of action. The antimicrobial properties of some plant-associated fungal endophyte-derived bioactive compounds have been given in Table 1.

**Table 1:** Fungal endophyte-derived antimicrobial compounds and their mode of action

Medicinal plants	Fungal endophytes	Metabolites	Anti-microbial effects	Mechanism	Reference
*Dioscorea zingiberensis*	*Rhexocercosporidium* sp. Dzf14	Rhexocercosporin D	Antibacterial	Rhexocercosporin D might disrupt bacterial membranes and assist colistin in the synergistic destruction of bacteria by disrupting membrane homeostasis	[115]
*Sophora tonkinensis*	*Xylaria* sp. GDGJ-77B	Xylarchalasin B	Antibacterial	^a^	[116]
*Gynostemma pentaphyllum*	*Chaetomium globosum* CGMCC 6882	Genistein combined polysaccharide (GCP)	Antibacterial	Depolarization of bacterial cell membrane, downregulation of Ca^2+^-Mg^2+^-ATPase on bacterial cell membrane, and upregulation of Ca^2+^ ions in cytoplasm	[117, 118]
*Glycyrrhiza glabra* Linn	*Phoma* sp.GG1F1	Thiodiketopiperazine derivatives	Antibacterial	Downregulation of bacterial transcription/translation process, staphyloxanthin production, and upregulation of Staphylococcal enterotoxin A (*sea*) gene	[96]
*Ginkgo* *biloba* L	*Penicillium cataractum* SYPF 7131	Penicimenolidyu A; Penicimenolidyu B; and Rasfonin	Antibacterial	Downregulation of protein FtsZ	[119]
Grapevine	*Alternaria alternata*	Cyclo(l-phenylalanine-trans-4-hydroxy-l-proline); cyclo(l-leucine-trans-4-hydroxy-l-proline); and cyclo(l-alanine-trans-4-hydroxy-l-proline)	Antifungal	Sporulation inhibition activity	[120]
*Hypericum perforatum* (St John’ Wort)	*Aspergillus* sp. TJ23	Aspemerodione	Antibacterial	Downregulation of protein PBP2a (penicillin-binding protein 2a)	[121]
*Quercus emoryi*	*Alternaria tenuissima* QUE1Se	Altertoxins	Antiviral	Downregulation of HIV1	[122]
*Heteropogon* *contortus*	*Chaetomium subaffine*	Phytosphingosine, hexadecasphinganine, andrographolide, and schaftoside	Antibacterial	Inhibition of bacterial cell wall biosynthesis, bacterial DNA, and protein biosynthesis	[123]
*Zingiber officinale* Rosc	*Paraconiothyrium* sp.	Danthron (1)	Antifungal, Antibacterial	^a^	[24]
*Ginkgo biloba* L	*Aspergillus* sp. IFB-YXS	Xanthoascin (2)	Antibacterial	^a^	[124]
*Anvillea garcinii* (Burm.f.) DC	*Fusarium chlamydosporium*	Fusarithioamide A (3)	Antibacterial and antifungal	^a^	[125]
*Garcinia dulcis* (Roxb.) Kurz	*Phomopsis* sp. PSU-D15	Phomoenamide (4)	antimycobacterial	^a^	[126]

^a^Metabolites isolated from fungal endophytes are not reported for their mode of action. This information is not in the study of research paper available so far in my knowledge

## Fungal Endophyte-Derived Compounds with Antioxidant Activities

An antioxidant is a chemical that when present in small amounts in comparison with the oxidizable substrate considerably slows or prevents its oxidation [127]. Antioxidants present in phenolic compounds can prevent free radical production and/or stop the progression of autoxidation. Plant extracts, which are typically employed for their flavoring properties, frequently contain high H-donating activity, which makes them particularly potent antioxidants [128–130]. Reactive oxygen species comprise a variety of oxygen radicals consisting of hydroxyl radical, peroxyl, and hydroperoxyl along with superoxide radical, in addition to nonradical oxidizing agents such as hydrogen peroxide, ozone, hypochlorous acid, and others which may be easily transformed to radicals. Fungal metabolites have the potency to scavenge ROS produced by metabolism in cells of plants or animals [131]. Fungal endophyte-derived bioactive compounds exhibiting antioxidant properties have been reported in prior studies [132–135]. A recent study elucidated the fermentation, separation, structural elucidation, and antioxidant effects of the fungal endophyte-derived compound. The fungal colonies were isolated from the host plant using potato dextrose agar medium to culture fungi for single-colony isolation and M1D medium to culture fungus for mass production of metabolites. Thin-layer chromatography was used to micro-fractionate fungal metabolites, which were then purified via the silica gel column chromatographic method. Structural characterization of fungal endophyte-derived bioactive compounds was performed by using techniques like NMR (nuclear magnetic resonance), EPR (electron paramagnetic resonance), HREIMS (high-resolution electron spray ionization mass spectrometry), and X-ray crystallography. *Pestalotiopsis microspora*, a fungal endophyte that survives in the *Terminalia morobensis* plant, generated the bioactive chemical isopestacin. This chemical showed antioxidant properties against superoxide as well as hydroxy free radicals [136]. An endophytic fungus *Cephalosporium* sp. IFB-E001 dwells inside the host plant *Trachelospermum jasminoides* and produced the bioactive compound graphislactone A. The antioxidant activities of the compound graphislactone A were found against DPPH (2,2-diphenyl-1-picrylhydrazyl) radicals, and Cu^2+^-induced LDL peroxidation having IC_50_ values of 9.6 mM and 7.3 mM respectively. It also exhibited a significant antioxidant effect against hydroxyl radicals and linoleic acid peroxidation [132]. The fungal endophyte *Acremonium* sp., derived from the *Garcinia griffithii* plant, produced the bioactive compound 3,5-dihydroxy-2,5-dimethyltrideca-2,9,11-triene4,8-dione. This compound exhibited potent antioxidant activity towards DPPH free radicals having an IC_50_ value of 10.8 µg/mL [137]. A fungal endophyte *Monascus purpureus*, dwelling in the root tissues of the host plant *Avicennia marina*, produced antioxidant pigments. The fungus’ methanolic extract produced the most extracellular colorants (647.87 mg. equivalent of Carmine per liter) and exhibited antibacterial as well as antioxidant activity. The colorants had been shown to be an antioxidant effect on ABT (2,2-azino-bis-3-ethylbenzothiazoline-6-sulphonic acid) radicals with an IC_50_ value of 14.42 µg/mL along with a TEAC (Trolox-equivalent antioxidant capacity) value with 0.571 µM Trolox/µg [138]. The ethyl acetate (EA) extract of *Fusarium foetens* AQF6, a fungal endophyte that survives in the host plant *Amentotaxus yunnanensis* H.L.Li, showed a scavenging % potential of 95.75 ± 1.1% and 85.66 ± 1.91% towards free radicals as well as hydroxyl radicals, respectively, at a concentration of 400 µg/ml. Fifteen compounds were isolated and characterized using the HPLC technique, with their chemical structures identified through HR-ESI–MS, 1D, and 2D NMR spectroscopy analyses from the crude extract of *Fusarium foetens* AQF6. Among the identified 15 compounds, a new sesquiterpene FUS (Fusafoetriol) and five known compounds namely phenylacetic acid, p-hydroxybenzaldehyde, 2-(4-hydroxyphenyl)ethanol, salicylic acid, and isoformononetin exhibited antioxidant activity against DPPH free radicals with IC_50_ values of 1.76 ± 0.32 Mm, 3.73 ± 0.39 mM, 1.65 ± 0.27 mM, 5.33 ± 0.06 mM, 0.95 ± 0.08 mM, and 1.02 ± 0.09 mM, respectively, and against hydroxyl radical with IC_50_ values of 0.95 ± 0.37 mM, 4.46 ± 0.86 mM, 0.36 ± 0.09 mM, 3.97 ± 0.11 mM, 1.92 ± 0.61 mM, and 1.14 ± 0.25 mM, respectively. The new compound fusafoetriol (FUS), at concentrations of 2 mM and 4 mM, showed yeast cell survival rates of 28.49% and 43.31%, respectively, against H_2_O_2_-induced oxidative stress [139].

### Fungal Endophyte-Derived Compounds with Cytotoxic and Anticancer Effects

Plants are the reservoir of anticancer compounds [140]. Even after several decades of study, cancer remains a significant worldwide health issue [141]. Anticancer compounds generally target the DNA and DNA-binding proteins of cells [142]. The anticancer characteristics of bioactive chemicals include anti-angiogenic, anti-migratory, and anti-proliferative activities [143]. Previous research demonstrated that fungal endophytes could serve as a promising source of anticancer compounds [144–147]. Another study demonstrated the fermentation process, separating the compound, determining its chemical structure, and assessing its cytotoxicity and potential anticancer effects. The fermentation of fungal endophyte was performed by using a potato dextrose broth medium to produce bioactive metabolites in large quantities. The study employed silica gel and flash reverse-phase chromatography techniques for bioactivity-guided fractionation of fungal metabolites. Structural elucidation of bioactive compounds had been identified by spectroscopy analysis like ^13^C NMR, ^1^H NMR, COSY (correlation spectroscopy), HMBC (heteronuclear multiple bond correlation), ROESY (rotating frame Overhauser effect spectroscopy), and IR (infrared) spectroscopy along with X-ray crystallography. The fungal endophyte *Pestalotiopsis microspora* was derived from the plant *Torreya taxifolia* and generated the bioactive compound torreyanic acid. The compound torreyanic acid appeared to be approximately 5–10 times comparatively more effective towards cell lines responsive to agonist of protein kinase C along with induced apoptotic cell death. It also exhibited cytotoxic activity towards 25 distinct cancerous cell lines with IC_50_ values ranges between 3.5 µg/mL (NEC—neuroendocrine carcinoma) and 45 µg/mL (A549). It can induce cell cycle arrest during the G_1_ stage of G_0_ synchronized cells at concentrations ranging between 1 and 5 µg/mL, based on the characteristics of the cell line [148]. An endophytic fungus *Aspergillus parasiticus* dwells inside the *Sequoia sempervirens* plant and produces two novel bioactive compounds namely sequoiatone A and sequoiatone B. The compounds sequoiatone A and sequoiatone B also showed selective inhibition of the growth of human tumor cells, along with a stronger effect observed towards breast cancerous cell lines. The maximum value of GI_50_ obtained for sequoiatone A and sequoiatone B was between 4 and 10 µM, indicating that these doses were effective in preventing tumor cell proliferation. The LC_50_ values, on the other hand, were larger than 100 µM, indicating that the chemicals were not very hazardous to the cells at growth inhibitory concentrations [149]. The cytotoxic compounds dicerandrols A–C were extracted through bioactivity-guided fractionation of the crude extract of a fungal endophyte *Phomopsis longicolla*, isolated from the *Dicerandra frutescens* plant [150]. The fungal endophyte *Talaromyces radicus*, derived from the plant *Catharanthus roseus*, produced potent compounds vincristine and vinblastine. Both these compounds had the ability to cause apoptotic cell death, making them useful in cancer therapy [151]. The crude metabolites of a fungal endophyte *Penicillium citrinum* Thom., derived from the plant *Jatropha heynei*, was shown to be cytotoxic in both A549 and MCF-7 cells having IC_50_ values of 280.7 µg/mL and 283.0 µg/mL, respectively [152]. Using the OSMAC approach and molecular networking, 12 chemicals were identified from an endophytic fungus *Talaromyces* sp. CY-3. These compounds include sambutoxin C, sambutoxin D, sambutoxin A, sambutoxin E, sambutoxin B, *N*-demethylsambutoxin, ( −)-sambutoxin, sambutoxin F, sambutoxin G, ilicicolin H, deoxyleporin B, and leporin B. The chemical structure of these compounds had been identified using 1D/2D-NMR, HR-ESI–MS, and ECD spectra analysis, along with the common biosynthesis path. These isolated compounds showed cytotoxic effects towards cancer cell lines of MDA-MB-435 (M.D. Anderson-Metastatic Breast-435) cells, MDA-MB-231 (M.D. Anderson-Metastatic Breast-231) cells, A549 cells, SNB19 cells, HCT116 cells, and PC-3 cells exhibiting IC_50_ concentrations ranging from 1.76 to 49.13 μM. The compound ilicicolin H was chosen for evaluating its mechanism of action against cancer. Ilicicolin H exhibited cytotoxic activities towards cancer cell lines such as MDA-MB-435, MDA-MB-231, SNB-19, HCT-116, A-549, H1703, 4T1, CT26, MC38, B16F10, Hepa1-6, and LLC cells with IC50 values of 6.34 ± 1.626 µM, 9.98 ± 0.025 µM, 14.59 ± 3.742 µM, 10.75 ± 0.907 µM, 27.90 ± 0.223 µM, 6.65 ± 0.062 µM, 13.84 ± 0.921 µM, 19.54 ± 0.638 µM, 19.55 ± 0.902 µM, 21.580 ± 0.950 µM, 19.570 ± 0.667 µM, and 5.78 ± 0.127 µM, respectively. Ilicicolin H exhibited G_0_/G_1_ phase cell cycle arrest through upregulation of the p53 along with p21/CyclinD1/Rb signaling pathways [153]. The anticancerous properties with their mode of action of some plant-associated fungal endophyte-derived bioactive compounds have been given in (Tables 2 and 3).

**Table 2:** Fungal endophyte-derived anticancer compounds with their in vitro effects and mechanism of action

Medicinal plants	Endophytes	Metabolites	Anticancer Effects	Mechanism	Reference
*Anoectochilus roxburghii*	*Fusarium concentricum*	Sambutoxin	Cytotoxicity towards HT29 cells as well as PC3 cells with IC_50_ concentrations of 7.60 μM and 4.99 μM, respectively	^a^	[154]
*Macrozamia communis*	*Penicillium* sp. MNP-HS-2	Mycophenolic acid methyl ester	Cytotoxicity towards cancer cell lines comprise L929, KB3.1, A549, A-431, PC-3, MCF-7, and SKOV-3 cells with IC_50_ values of 0.2 μM, 0.2 μM, 0.4 μM, 0.1 μM, 0.4 μM, 0.1 μM, and 0.1 μM, respectively	^a^	[155]
*Gynochthodes officinalis*	*Paramyrothecium roridum*	Pararorine A	Cytotoxicity towards cancer cell lines such as SF-268, MCF-7, HepG2, and A549 with IC_50_ concentrations ranges between 1.69 and 8.95 μM	Pararorine A inhibited cancer cell growth in HepG2 cells by stimulating cytochrome C levels, causing a cell cycle arrest, along with triggering apoptosis by upregulating expression of proteins like JNK as well as Bax	[156]
*Ruprechtia salicifolia*	*Emericella nidulans* ATCC 38163	Emestrin	Cytotoxicity towards Huh-7 (hepatic carcinoma) cells, A-549 (lung cancer) cells, and Caco2 (colon cancer) cells with IC_50_ values of 4.89 µM, 6.3 µM, and 9.28 µM, respectively	Emestrin potentially inhibits the proliferation of Huh-7 (hepatocellular carcinoma) cells by targeting G_1_/S cell cycle arrest and triggering apoptosis through upregulation of Bax, p53, and caspase-9, while downregulating bcl-2 proteins	[157]
*Pseudostellaria heterophylla*	*Talaromyces primulinus* WZ-883	PP-R (7-(2-hydroxyethyl)-monascorubramine)	Cytotoxicity towards various colorectal cancerous cell lines such as HCT116 cells, HT29 cells, HCT115 cells, and SW620 cells with IC_50_ values of 4.17 ± 0.11 µM, 9.38 ± 0.24 µM, 8.12 ± 0.16 µM, and 6.95 ± 0.45 µM, respectively	PP-R effectively suppresses the proliferation of HCT116 cells by triggering autophagy, as well as apoptosis, potentially through the MAPK along with mTOR signaling pathways, suggesting its potential for colorectal cancer treatment	[158]
*Mentha pulegium*	*Stemphylium globuliferum*	Altersolanol A (5)	Effective cytotoxicity towards L5178Y cells at a concentration of 10 µg/mL along with strong kinase inhibition activity against Aurora-B as well as CDK4/CycD1 with IC_50_ values of 6.7 µg/mL and 0.64 µg/mL, respectively	Downregulation of NF-κB transcriptional activity	[159, 160]
*Astragalus fruticosus*	*Aspergillus flavus* ER	Camptothecin	Cytotoxic towards HEPG-2 cells, MCF7, and HCT29 cells with IC_50_ values of 0.9 mM, 1.2 mM, and 1.35 mM, respectively	Camptothecin suppressed proto-oncogenes along with anti-apoptotic genes and promoted the expression of pro-apoptotic as well as smaller ribosomal protein genes, along with p53 mark genes	[161, 162]
*Rheum emodi*	*Polyporales* sp.	Emodin (Rz) (6)	Effective cytotoxic effect towards various cancerous cell lines such as A549, NCI-H322, and Colo-205 with both testing concentrations 70 µM and 100 µM	Emodin’s anticancer activities were demonstrated through cell cycle arrest during the G_1_ and G_2_/M phases	[163]
*Cassia fistula* L	*Penicillium sclerotiorum*	Hexadecanoic, oleic along with benzoic acid	Cytotoxic effect towards HeLa cells, A549, A431, and U251 cells with IC_50_ values of 7.75 μg/ml, 10 μg/ml, 20 μg/ml, and 32 μg/ml, respectively	Induce arrest of the cell cycle in the S phase as well as in G_2_/M phase	[164]
*Trichocolea tomentella*	*Penicillium concentricum*	3-hydroxybenzenemethanol	Cytotoxic effect towards HeLa, PC-3, and DU-145 cells with IC_50_ values of 13.7 ± 0.8 μmol/l, 3.6 ± 0.8 μmol/l, and 5.6 ± 0.5 μmol/l, respectively	Inhibition of NF-KB activity and induced mitochondrial membrane potential (MTP) damage	[165]
*Trichocolea tomentella*	*Penicillium concentricum*	2-bromogentisyl alcohol	Cytotoxic effect towards HeLa, PC-3, DU-145, and MDA-MB-231 with IC_50_ values of 3.1 ± 0.3 μmol/l, 2.7 ± 0.3 μmol/l, 2.7 ± 0.01 μmol/l, and 1.8 ± 0.3 μmol/l, respectively	Induction of mitochondrial membrane potential (MTP) damage	[165]
*Trichocolea tomentella*	*Penicillium concentricum*	Epoxydon (7)	Cytotoxic towards DU-145 (IC_50_ 1.2 ± 0.6 μmol/l)	Cell cycle arrest in the G2 phase along with induction of MTP damages	[165]
*Piper nigrum*	*Colletotrichum gloeosporioides*	Piperine (8)	Cytotoxic active towards A549 cells exhibiting an IC_50_ value of 122 μg/ml	It induces a p53-dependent arrest of the cell cycle during the G2/M phase, along with caspase-9-mediated intrinsic apoptotic pathways, with an increase in the ratio of the Bax/Bcl-2	[166, 167]
*Sinopodophyllum hexandrum*	*Pestalotiopsis adusta*	Pestalustaines A and B	Cytotoxic effect towards HeLa, HCT116, and A549 cells with IC_50_ values that range between 21.01 and 55.43 µM	^a^	[168]
*Capsicum annum*	*Alternaria alternata*	Alternariol-10-methyl ether	Cytotoxic effect towards HL-60 and A431 cells with IC_50_ values of 85 µM and 100 µM, respectively	Induce apoptosis, along with mitochondrial-membrane potential (MTP) damage	[169]
*Ephedra fasciculata*	*Fusarium oxysporum* EPH2R_AA_	Beauvericin	Cytotoxic effect towards cell line of NCI-H460 (IC_50_ 1.41 µM); MIA Pa Ca-2 (IC_50_ 1.66 µM); MCF-7 cells (IC_50_ 1.81 µM) along with SF-268 cells (IC_50_ 2.29 µM) respectively	Showed antiangiogenic effect by disrupting endothelial cell network development	[170]
*Mimosops elengi* (bakul)	PM0651480	Ergoflavin	Cytotoxic activity towards cancerous cell lines of ACHN (IC_50_ 1.2 ± 0.20 µM), H460 (IC_50_ 4.0 ± 0.08 µM), Panc1 (IC_50_ 2.4 ± 0.02 µM), HCT116 cells (IC_50_ 8.0 ± 0.45 µM), along with Calu1 (IC_50_ 1.5 ± 0.21 µM) respectively	Antiproliferative	[171]
*Adenophora axilliflora*	*Chaetomium* sp. IFBE015	Chaetominine (9)	Cytotoxic activity towards K562 and SW1116 cells with IC_50_ values of 21.0 nM and 28.0 nM, respectively	Upregulation of p‑ATR and Chk1; downregulation of cdc25A causes arrest of the cell cycle in the S-phase	[172]
*Tabebuia rosea*	*Aspergillus* TRL1	Pulchranin A	Cytotoxicity towards cancer cell lines MCF-7 (IC_50_ 63 µg/mL); Hep-G2 (IC_50_ 80 µg/mL); and HCT (IC_50_ 91 µg/mL) respectively	Downregulation of cyclin-dependent kinases such as CDK1, CDK2, and CDK4	[173]
*Chaetomorpha media*	*Chaetomium globosum*	Chrysin (10)	Cytotoxicity towards MCF-7 cells exhibiting an IC_50_ value of 49.0 ± 0.6 µM	Induced apoptosis, ROS generation, and arrest of the cell cycle in the G1 phase, along with damage of the mitochondrial membrane potential	[174]
*Catharanthus roseus*	*Eutypella* spp. — CrP14	Vincristine	Cytotoxicity towards A431 cells having an IC_50_ value of 4.8 ± 0.33 μg/ml	Induced apoptosis in A431 cells by ROS production and mitochondrial membrane potential damage	[175]
*Taxus celebica*	*Fusarium solani*	Paclitaxel	Effective cytotoxic activity towards various cell lines such as JR4-Jurkat, HepG2, HeLa, Ovcar3, and T47D cells with IC_50_ values of 0.006 ± 0.0003 μM, 0.1 ± 0.02 μM, 0.008 ± 0.001 μM, 0.2 ± 0.05 μM, and 0.005 ± 0.001 μM, respectively	Arrest of the cell cycle during the G1 phase, induced apoptosis via caspase 10-mediated cascades, along with mitochondrial-membrane potential damage	[176, 177]
*Phyllanthus niruri* L	*Curvularia geniculata* L	2-methyl-7-phenylindole	Cytotoxic activity towards cell line HepG2 having an IC_50_ value of 62.23 μg/mL	Induced apoptosis by ROS generation, along with loss of mitochondrial membrane potential	[178]
*Dysoxylum binectariferum* Hook.f	*Fusarium proliferatum*	Rohitukine	Significant cytotoxicity toward HL-60 cells along with Molt-4 cells having GI_50_ values of 10 μg/ml and 12 μg/ml, respectively, and also exhibited Cdk2/A along with Cdk9/T1 kinase inhibition activity exhibiting IC_50_ values of 7.3 μM and 0.3 μM, respectively	Induced apoptosis through MAPK-mediated apoptotic pathway, along with ROS generation and activation of p53 protein (pro-apoptotic protein), as well as decreased expression of Bcl-2 protein (anti-apoptotic protein)	[179-181]

^a^Metabolites isolated from fungal endophytes are not reported for their mode of action. This information is not in the study of research paper available so far in my knowledge

**Table 3:** In vivo study of fungal endophyte extracts with anticancer properties

Medicinal plants	Endophytes	Extract	Anticancer activity	Dose/duration/LD_50_	Mechanism of action	Reference
*Ziziphus mauritiana*	*Trichoderma viride*	Ethyl acetate extract	Reduction in cervical intraepithelial neoplasia (CIN) squamous cell size along with reduced inflammation of stroma in Wistar albino rat with cervical cancer	2000 mg/kg body for oral toxicity	The level of cancer antigen significantly reduced at a concentration of 40 mg/kg body weight	[182]
*Taxus brevifolia*	*Fusarium solani*	Fungal taxol (FS)	Inhibitory effect on cancer cell proliferation in A549	Sub-acute oral administration of fungal taxol up to 500 mg/kg for a period of 28 days in Wistar rats	Cell cycle arrest at S and G2/M phase, induced ROS generation and extrinsic and intrinsic pathway-mediated apoptosis in A549 cell	[183]
*Commiphora wightii*	*Cladosporium* sp. (MycoAuNPs)	Aqueous extract	Antitumor	MycoAuNPs were evaluated for acute oral toxicity using Swiss Albino mice with a dose of 2000 mg/kg bwt over 14 days	Induction of ascite cells, decrease in the peritoneal fluid secretion by inhibition of neovascularization in the peritoneum	[184]
*Catharanthus roseus* (L.) G. Don	*Mucor* sp.	Chloroform extract	Acute toxicity	LD_50_ > 5000 mg/kg	Reduction in aberrant crypt foci (ACF)	[185]
*Aegle marmelos*	*Curvularia australiensis* FC2AP	Dimer of epicatechin (DoE)	Antiangiogenesis	The survival dose for albino mice was determined to be 1250 mg/kg bwt, while the lethal dose was observed at 1500 mg/kgbwt	Upregulation of catalase, superoxide dismutase, glutathione peroxidase, and glutathione enzyme activity	[186]

## Fungal Endophyte-Derived Compounds with Antidiabetic Activity

In diabetes, there is a metabolic dysregulation characterized by increased gluconeogenesis and reduced glycolysis in the liver, along with decreased glycogen synthesis. Additionally, lipid metabolism is also altered, leading to increased lipolysis and fatty acid production [187]. The anti-diabetic effect is demonstrated through the regulation of metabolic processes in the liver and kidney [188, 189]. Some antidiabetic compounds like metformin, insulin, and PPAR (peroxisome proliferator-activated receptor) agonists may affect the metabolic pathways by reducing gluconeogenesis, increasing glycolysis and glycogen production, or modulating lipid metabolism. Inhibitors of SGLT2 and metformin may hinder glucose reabsorption as well as decrease renal gluconeogenesis, respectively [190]. Furthermore, the experimental study suggests that during diabetes, the oxidative stress and inflammation are elevated in the kidney. However, anti-diabetic drugs having antioxidant and anti-inflammatory characteristics may help to reduce these effects [191, 192]. Antidiabetic compounds exhibited inhibition activity of enzymes alpha-glucosidase and alpha-amylase [193–195]. An endophytic fungal strain MEXU 27095, dwelling inside the plant host *Hintonia latiflora*, produced thielavin A, thielavin J, and thielavin K and exhibited concentration-dependent inhibition of *Saccharomyces cerevisiae*-derived α-glucosidase (αGHY) enzyme activity having IC_50_ concentrations of 23.8 μM, 15.8 μM, and 22.1 μM, respectively. The in vivo study showed that thielavin K had α-glucosidase inhibitory actions as shown by its strong antihyperglycemic activity in normal and nicotinamide-streptozotocin–induced diabetes-prone mice via the oral sucrose–tolerant experiment at dosages of 3.1, 10.0, and 31.6 mg/kg bwt. Furthermore, it demonstrated modest hypoglycemic action in diabetic mice at a dosage of 10 mg/kg bwt[196]. A fungal endophyte *Xylariaceae* sp. QGS 01, that survives in the tissue of the stem of the plant *Quercus gilva* Blume, generated an anti-diabetic chemical 8-hydroxy-6,7-dimethoxy-3-methylisocoumarine which exhibited significant α-glucosidase inhibition activity with a concentration of 41.75 μg/mL as the IC_50_ [197]. An endophytic fungus *Fusarium equiseti* derived from the *Gymnema sylvestre* plant produced the potent bioactive compound mycosterol that showed anti-diabetic properties. The endophytic fungus *Fusarium equiseti* crude extract showed inhibition activity of enzymes α-amylase along with α-glucosidase having IC_50_ values of 4.22 ± 0.0005 µg/mL and 69.72 ± 0.001 µg/mL, respectively [198]. A fungal endophyte *Nigrospora sphaerica* BRN 01 (NEE) that dwells in the leaf of the plant *Bauhinia purpurea* L. produced several bioactive compounds. The ethyl acetate crude extract of NEE exhibited significant inhibition activity of enzyme α-glucosidase having an IC_50_ concentration of 0.020 ± 0.001 mg/mL [199]. Yu et al. explore the potential antidiabetic effects of a new compound phomopamide A, characterized by a pentadepsipeptide structure. This compound comprises two phenylalanine units, one valine unit, one leucine unit, and one 2-hydroxyoctanoic acid unit. It is sourced from a fungal endophytic genus, *Diaporthe* sp., found within the host plant *Artemisia argyi*. Phomopamide A was found to have no cytotoxicity effect on cancer cell lines such as SF-268 cells, MCF-7 cells, HepG-2 cells, and A549 cells. However, phomopamide A demonstrated significant inhibition of α-glucosidase activity having an IC_50_ concentration of 62.35 ± 10.67 μM, in comparison with standard drug acarbose with an IC_50_ concentration of 154.1 ± 5.46 μM [200]. The antidiabetic properties with their mode of action of some plant-associated fungal endophyte-derived bioactive compounds have been given in Table 4.

**Table 4:** Some other fungal endophyte-derived compounds with anti-diabetic effects

Medicinal plants	Endophytes	Bioactive compounds	Antidiabetic activity	Mechanism	Reference
*Sinomenium acutumn*	*Pestalotiopsis palmarum*	Pestalotiophthalide A	The inhibitory activity of α-glucosidase has an IC_50_ concentration of 512.4 μM	It establishes hydrogen bond interaction with Asp87, Arg348, Arg400, and Met321 amino acid residues found within the active site of the α-glucosidase protein	[201]
*Boswellia sacra*	*Aureobasidium pollulan* BSS6	Methyl-5-docosenoate	The inhibitory activity of α-glucosidase has an IC_50_ concentration of 23.3 µM	π-Alkyl type of interactions with the crucial residues of amino acids Asp214 and Glu276 constitute the catalytic unit of the enzyme α-glucosidase	[202]
*Acanthus ilicifolius* L	*Epicoccum nigrum* SCNU-F0002	Epicocconigrone A (11)	The inhibitory activity of α-glucosidase has an IC_50_ concentration of 32.3 µM	^a^	[203]
*Juniperus polycarpos*	*Penicillium canescens*	1,2,3,5,6-pentahydroxy-8-methylxanthone, 1,3,5,6-tetrahydroxy-8-methylxanthone, along with 1,6-dihydroxy-3-methoxy-8-methylxanthone	The inhibitory activity of α-glucosidase has IC_50_ values ranging between 38.80 ± 1.01 and 75.20 ± 1.02 µM	1,2,3,5,6-pentahydroxy-8-methylxanthone (mixed inhibitor); 1,3,5,6-Tetrahydroxy-8-methylxanthone (competitive inhibitor), 1,6-dihydroxy-3-methoxy-8-methylxanthone (non-competitive inhibitor)	[204]
*Kandelia candel*	*Pestalotiopsis neglecta*	Neglectine A (15)	Protein tyrosine phosphatase (PTP) inhibition effect towards SHP1, CDC25B, and PTP1B, having IC_50_ concentrations of 17.1 μg/mL, 24.0 μg/mL, and 6.7 μg/mL, respectively	In type II diabetes, PTP1B regulates insulin signaling by dephosphorylating insulin receptor substrate (IRS). By inhibiting PTP1B, the activity of IRS is increased, leading to enhanced insulin signaling and improved glucose uptake by cells	[205]
*Lobophytum crassum*	*Scedosporium apiospermum* F41-1	Scequinadoline D (12)	Triglyceride-promoting effect toward cell line 3T3-L1 with an EC_50_ value of 0.27 ± 0.03 μM	Activate PPARγ pathway by stimulation of mRNA expression of PPARγ, AMPKα, and C/EBPα, LXRα, and SCD-1, and FABP4	[206]
*Hypericum perforatum* (St John’ Wort)	*Aspergillus* sp. TJ23	Asperpyridone A (13)	Significant glucose uptake activity towards the liver HepG2 cells	Activation of PCK2 and FGF21	[207, 208]
*Cucumis sativus*	*Paecilomyces formosus* LHL10	YW 3548, and paecilodepsipeptide A	The inhibitory activity of α-glucosidase has IC_50_ concentrations of 61.80 ± 5.7 µg/mL and 75.68 ± 6.2 µg/mL, respectively	^a^	[209]
*Quercus gilva* Blume	*Xylariaceae* sp. QGS 01	8-hydroxy-6,7-dimethoxy-3-methylisocoumarine	The inhibitory activity of α-glucosidase has an IC_50_ concentration of 41.75 μg/mL	^a^	[197]
*Ficus religiosa*	*Dendryphiom nanum* (Nees) S. Hughes	Herbarin (14)	Significant glucose uptake activity in rat skeletal muscle having an EC_50_ concentration of 0.80 ± 0.090 μM	^a^	[192]
*Hintonia latiflora*	MEXU 27905	Thielavin A, thielavin J, and thielavin K	The inhibitory activity of α-glucosidase has IC_50_ concentrations of 23.8 µM, 15.8 µM, and as 22.1 µM, respectively	Thielavins interact with homologous α-GHBs along with α-GHY, which have PDBs of 3A4A, with active pockets adjacent to the maltose and isomaltose catalytic sites, respectively	[196]
*Acacia nilotica*	*Aspergillus awamori*	Antidiabetic peptide	Inhibition of α-amylase along with α-glucosidase activity with IC_50_ values of 3·75 and 5·625 μg/ml respectively	^a^	[210]

^a^Metabolites isolated from fungal endophytes are not reported for their mode of action. This information is not in the study of research paper available so far in my knowledge

## Fungal Endophyte-Derived Compounds with Antimalarial Activity

**Fig. 3:**
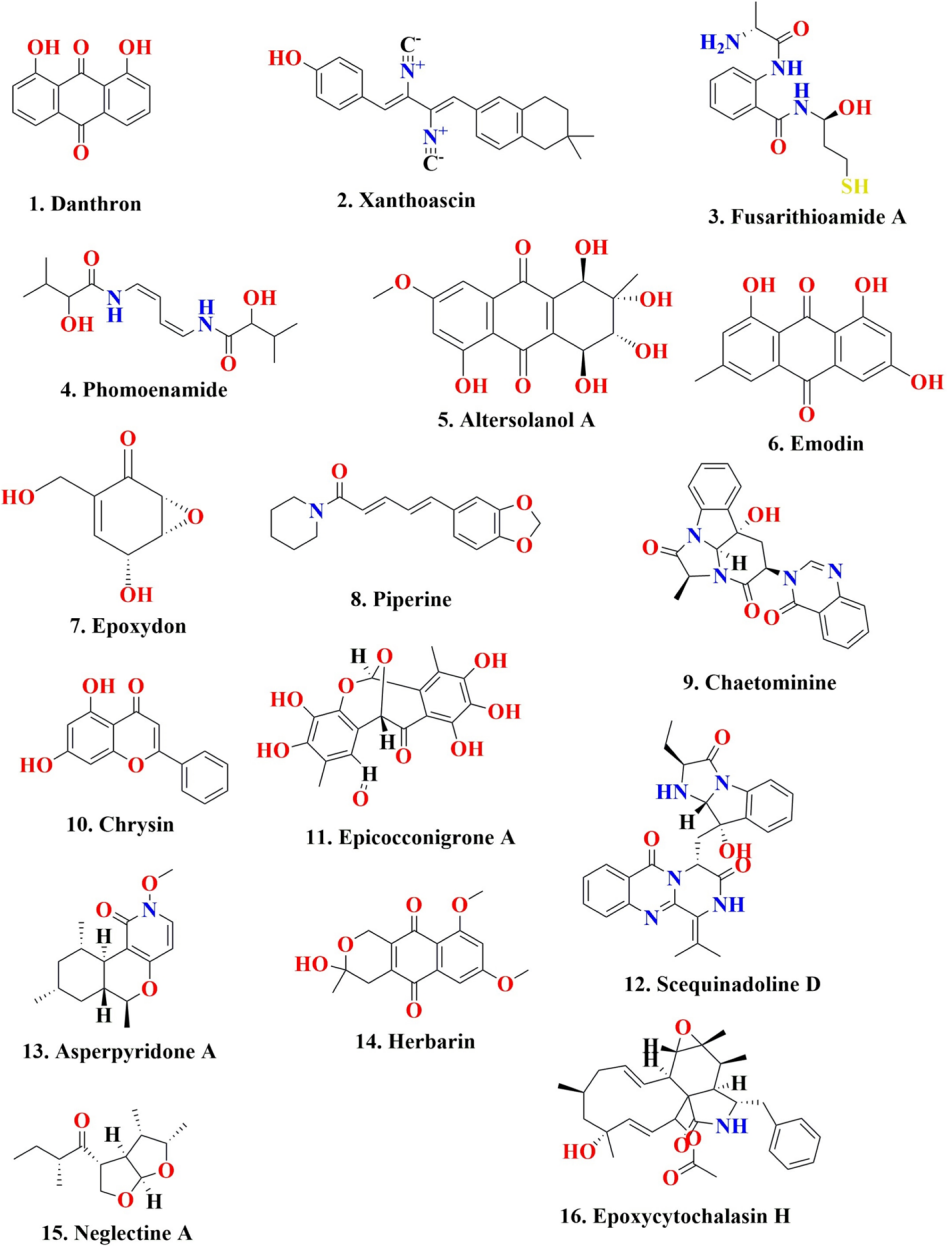
Elucidates the chemical structure of fungal endophytes derived biologically active compounds

Antimalarials are a class of chemical compounds, often derived from natural sources, useful for the treatment or prevention of malaria. They specifically target the malarial parasite and are effective against different stages of its life cycle. Antimalarials are commonly used to protect children and pregnant women, who are particularly vulnerable to the disease [211]. Plants are the natural sources of bioactive compounds with antimalarial activity [212–214]. The malarial parasite primarily derives its energy through anaerobic respiration, and the key enzyme in this process is LDH (lactate dehydrogenase), which converts pyruvate to lactate and generates NAD^+^. Consequently, LDH has recently become an alternative target for the malarial illness treatment [215]. Recently, small distinct organic molecules have been synthesized to serve as LDH inhibitors and their use in future drug development to combat malaria [216]. Some specific inhibitors that inhibit parasite LDH enzyme activity have been explored as a potent new antimalarial drugs [217]. Interfering with heme interaction towards histidine-rich protein-2 (HRP2) offers a promising antimalarial strategy to inhibit the growth of malarial parasites through disrupting the heme-HRP2 interaction. The inhibition of Heme detoxification in malarial parasites leads to accumulation of free heme in toxic form, which causes death of parasites [218]. A couple of studies demonstrated that natural compounds exhibited inhibitory effect to disrupt the interaction between heme-hrp2 complex [214, 219]. Similarly, a peptide and their cocktails also have been exhibiting similar activity to interfere with the development of the heme-hrp2 interaction [220]. These researches open up a new area of exploration into bioactive compounds and peptides as inhibitors of heme-hrp2 binding. Such discoveries might provide the way for alternative drugs against malaria in the pharmaceutical sector [221, 222]. A fungal endophyte *Pullularia* sp. BCC 8613 survived in the host plant *Culophyllum* sp. and produced four bioactive compounds pullularins A–D. The compound pullularin A exhibited antimalarial action towards *Plasmodium falciparum* K1 having an IC_50_ concentration of 3.6 μg/mL [223]. A fungal endophyte *Xylaria* sp. derived from the host plant *Sandoricum koetjape* generated two novel compounds 2-chloro-5-methoxy-3-methylcyclohexa-2,5-diene-1,4-dione and xylariaquinone A demonstrated antimalarial action towards *Plasmodium falciparum* K1 having IC_50_ concentrations of 1.84 µM and 6.68 µM, respectively [224]. Bioactive metabolites were identified by LC-HR-MS-based metabolomics, and a multivariate approach along with the in silico data analysis was performed to screen antimalarial active metabolites. A total of 11 endophytic fungi were derived from tissues of the leaf and stem of the plant *Artemisia annua* exhibited antimalarial activity. The extracts produced by the three *Penicillium* strains showed significant antimalarial activity towards pathogenic *Plasmodium falciparum* strains, having an IC_50_ concentration ranging between 1.1 and 3.3 µg/mL; *Talaromyces* strains with IC_50_ values of 7.6 ± 2.4 and 9.9 ± 2.1 µg/mL, followed by *Aspergillus terreus*-derived extract with the least antimalarial action having an IC_50_ concentration of 351.1 µg/mL. The in silico study revealed that the endophyte extract had antimalarial compounds like Emodin (6) (Fig. 3) and Physcion [225]. A fungal endophyte *Aspergillus niger* 58 survived in the tissue of the host plant *Terminalia catappa* and generated two bioactive compounds, namely, flavasperone and aurasperone A. The compound aurasperone A showed strong antimalarial activity towards *Plasmodium falciparum* 3D7 having an IC_50_ concentration of 4.17 μM along with antimalarial action towards *Plasmodium falciparum* INDO (*Pf*INDO) having an IC_50_ concentration of 3.08 μM, respectively. The crude extract of *Aspergillus niger* 58 showed antimalarial action towards *Plasmodium falciparum* 3D7 (Pf3D7) strain having an IC_50_ concentration of 4.03 μg/mL. Two fractions of fungal crude, namely, RP-HPLC F17 and RP-HPLC F18, also showed antimalarial action towards *Plasmodium falciparum* 3D7 (*Pf*3D7) strain having IC_50_ concentrations of 0.09 μg/mL and 0.1 μg/mL, respectively [226]. Violaceoid A, a hydroquinone derivative sourced from a fungal endophyte *Aspergillus aculeatus*, showed antimalarial activity towards the *Plasmodium falciparum* (K1) strain, having an IC_50_ concentration of 9.62 μM [227]. The antimalarial properties of some plant-associated fungal endophyte-derived bioactive compounds have been given in Table 5.

**Table 5:** Fungal endophyte derived bioactive compounds with antimalarial properties

Medicinal plants	Endophytes	Antimalarial compounds	Antiparasitic and antimalarial activity	Reference
*Toona sureni*	*Periconia pseudobyssoides* K5	Diketopiperazine cyclo-(S-Pro-R-Leu)	Inhibitory action towards Heme polymerization exhibiting IC_50_ concentrations of 9.89 ± 0.24 mmol/L	[228]
*Sandoricum koetjape*	*Xylaria* sp.	2-chloro-5-methoxy-3-methylcyclohexa-2,5-diene-1,4-dione, along with xylariaquinone A	Antimalarial action towards K1 strain of *Plasmodium falciparum* exhibiting IC_50_ concentrations of 1.84 µM and 6.68 µM, respectively	[224]
*Triticum* sp	*Nigrospora oryzae* CF-298113	Pipecolisporin	Antimalarial action towards *Plasmodium falciparum* and *Trypanosoma cruzi*	[229]
*Vellozia gigantea*	*Diaporthe miriciae*	Epoxycytochalasin H (16)	*Plasmodium falciparum*-specific antimalarial activity having an IC_50_ about 3.5 times less in comparison with chloroquine	[230]
*Mentha longifolia* L	*Fusarium* sp.	Fusaripeptide A	Antimalarial action against D6 strain of *Plasmodium falciparum* having an IC_50_ concentration of 0.34 μM	[231]
*Tectona grandis* L	*Phomopsis* sp. BCC 1323	Phomoxanthone A, along with phomoxanthone B	Antimalarial action towards K1 strain of *Plasmodium falciparum* having IC_50_ concentrations of 0.11 μg/ml and 0.33 μg/ml respectively	[232]
*Tinospora crispa* L	BB4 strain	7-hydroxy-3,4,5-trimethyl-6-on-2,3,4,6-tetrahydroisoquinoline-8-carboxylic acid	Anti-malarial action towards 3D7 strain of *Plasmodium falciparum* having an IC_50_ concentration of 0.129 μM	[233]
*Garcinia nigrolineata*	PSU-N24 strain	3-(2-Hydroxypropyl)benzene-1,2-diol	Anti-malarial activity towards 3D7 strain of *Plasmodium falciparum* having an IC_50_ concentration of 6.68 μg/ml	[234]
*Torreya taxifolia*	*Nemania* sp. UM10M	19,20-epoxycytochalasin C, 19,20-epoxycytochalasin D, along with 18-deoxy-19,20-epoxy-cytochalasin C	Anti-malarial action towards chloroquine-sensitive (D6)-strain having IC_50_ concentrations of 0.07 µM, 0.04 µM, and 0.56 µM respectively, and also against chloroquine-resistant (W2)-strain having IC_50_ concentrations of 0.05 µM, 0.04 µM, and 0.19 µM, respectively	[235]
*Crassocephalum crepidioides*	*Geotrichum* sp.	7-butyl-6,8-dihydroxy3(R)-pent-11-enylisochroman-1-one, as well as 7-butyl-6,8-dihydroxy-3(R)-pentylisochroman-1-one	Anti-malarial action towards K1 strain of *Plasmodium falciparum* having IC_50_ concentrations of 4.7 µg/mL and 2.6 µg/mL respectively	[236]
*Siparuna* sp.	*Xylaria* sp. Grev	( +)-phomalactone, and 5-hydroxymellein	Anti-malarial action towards *Plasmodium falciparum* having IC_50_ concentrations of 13 µg/mL and 19 µg/mL, respectively	[237]
*Stemona* sp.	*Exserohilum rostratum*	Monocerin (17) and 11-hydroxymonocerin	Anti-malarial action towards K1 strain of *Plasmodium falciparum* having IC_50_ concentrations of 0.68 μM and 7.70 μM, respectively	[238]
*Asterogyne martiana*	*Chalara alabamensis*	Viridiol (18)	Anti-malarial action towards *Plasmodium falciparum* having an IC_50_ concentration of 15.52 μM	[239]
*Melaleuca quinquenervia*	*Pestalotiopsis* sp.	Pestalactam A and pestalactam B	Anti-malarial activity towards *Plasmodium falciparum* (chloroquine-sensitive/chloroquine-resistant) with IC_50_ concentrations of 16.2/41.3 μM and 20.7/36.3 μM respectively	[240]
*Vanilla albidia*	*Phomopsis archeri*	Phomoarcherin B (19)	Anti-malarial activity towards *Plasmodium falciparum* having an IC_50_ concentration of 2.05 μM	[241]
*Vochysia guatemalensis*	*Codinaeopsis gonytrichoides*	Codinaeopsin (20)	Anti-malarial activity towards 3D7 strain of *Plasmodium falciparum* having an IC_50_ concentration of 4.7 μM	[242]
*Culophyllum* sp.	*Pullularia* sp. BCC 8613	Pullularin A and pullularin B	Antimalarial action towards *Plasmodium falciparum* having IC_50_ concentrations 4.63 μM and 4.17 μM, respectively	[223]

## Fungal Endophyte-Derived Compounds with Neuroprotective Activity

Neuroprotective chemicals are molecules that protect neurons in the brain and central nervous system for healthy functioning. They can help to prevent or reduce the effects of neurodegenerative diseases, injuries, and stressful circumstances [243]. Mitochondridrial dysfunction becomes a major cause of neurodegenerative circumstances [244]. Neuroprotective compound exhibits antioxidant, anti-inflammatory, and growth-promoting characteristics that contribute to neuroprotection [245–248]. Phytochemicals exhibit multitargeted characteristics with a biological system to combat neuronal dysfunction [249]. Phytochemicals exert a neuroprotective effect through regulating numerous neurotransmitters and their receptors of neurons in the brain for appropriate functioning [250, 251]. Chrysogenamide A was obtained from the fungal endophyte *Penicillium chrysogenum*, which dwells inside the root tissue of the host plant *Cistanche deserticola* Y. C. Ma. The compound chrysogenamide A exhibited a neuroprotective action towards oxidatively damaged SH-SY5Y cells [252]. An endophytic fungus *Penicillium citrinum* that survives in the tissue of plant host *Bruguiera gymnorrhiza* produced bioactive compounds namely (Z)−7,4′-dimethoxy-6-hydroxyl-auraone-4-O-β-glucopyranoside, along with (1S,3R,4S)−1-(4′-hydroxyl-phenyl)−3,4-dihydro-3,4,5-trimethyl-1*H*−2-benzopyran-6,8-diol. The compound (Z)−7,4′-dimethoxy-6-hydroxy-aurone-4-O-β-glucopyranoside demonstrated considerable neuroprotective efficacy in MPP^+^-induced oxidative stressed PC12 cells by increasing mitochondrial membrane potential, decreasing DNA fragmentation, and inhibiting caspase-3 and caspase-9 cascades [253]. Fungal endophyte *Colletotrichum* sp. JS-0367, derived from the plant *Morus alba*, produces the compound evariquinone (21) which exhibited a neuroprotective action towards oxidative damaged HT22 (hippocampal neuronal) cells via downregulation of intracellular ROS production, Ca^2+^ influx, and glutamate-induced phosphorylation of JNK, ERK_1/2_, and p38 proteins [254]. *Neosartorya fischeri* JS0553, an endophytic fungus, produces bioactive compound Fischerin (23) which exhibited a neuroprotective effect against oxidative damaged HT22 (hippocampal neuronal) cells via downregulation of intracellular ROS production, Ca^2+^ influx, and glutamate-induced phosphorylation of JNK, ERK_1/2_, and p38 proteins [255]. A fungal endophyte *Nigrospora oryzae*, which survives in the *Tinospora cordifolia* plant, produces a bioactive compound quercetin. The *Nigrospora oryzae* (GL15) crude extract exhibited a potent neuroprotective effect via the inhibition of acetylcholinesterase (AChE) activity in scopolamine (SCO)-induced mice [256]. Fan et al. investigated the neuroprotective features of undescribed pyrone derivative compounds produced by the fungal endophyte *Nigrospora oryzae*, which survive in the root tissue of the host plant *Taxus chinensis* var. *mairei*. In order to determine the structures and absolute configurations of the six known compounds, namely, solanapyrones A–C and solanapyrones E–G and the uncharacterized pyrones, solanapyrones U–Z, and precursor-like compounds prosolanapyrones A–B, a thorough spectroscopic analysis was performed along with the adapted Mosher’s process along with ICD (induced circular dichroism) spectrum analysis. All undescribed compounds underwent assessment for nerve growth factor (NGF) expression using HEK-293 T cells and bMSCs (bone marrow mesenchymal stem cells). Solanapyrones A–B and a novel pyrone, solanapyrone U, exhibited superior neuroprotective effects compared to the standard drug Clenbuterol in stimulating NGF secretion from bMSCs [257].

A study transformed common ginsenosides in American ginseng stems and leaves (AGSL) into rare ginsenosides using endophytic fungal fermentation. The fermented extract showed higher saponin content and significantly inhibited Aβ42 concentration and β-secretase activity, indicating potential as a therapeutic or nutritional treatment for Alzheimer’s disease [258]. Amyotrophic lateral sclerosis (ALS) involves toxic SOD1 mutations. Phialomustin-B, a fungal metabolite, reduces SOD1 aggregation by binding at the dimer interface, offering therapeutic potential for familial ALS (fALS) [259]. Neuroprotection is crucial against damage from neurodegenerative diseases like Alzheimer’s and Parkinson’s [260–262]. Endophytic fungi produce bioactive compounds that support growth factors, enhance antioxidant defenses, and reduce neuroinflammation. Evaluated through enzymes, cell lines, and in vivo models, these compounds show promise, though clinical trials are needed to confirm their neuroprotective efficacy and safety [263]. A recent study identified an endophytic fungus, *Fusarium* sp., from tea leaves that inhibit α-synuclein aggregation by reducing oxidative stress and oligomerization, showing potential for Parkinson’s disease therapy [264]. Another study identified *Aspergillus niveus* Fv-er401 from *Foeniculum vulgare* roots with anticholinesterase activity. Isolated compounds, including terrequinone A and citrinin, and others showed strong AChE and BuChE inhibition, suggesting potential for Alzheimer’s drug development [265]. In addition, a study identified endophytic fungi from *Catharanthus roseus* with acetylcholinesterase (AChE) inhibitory activity. Active compounds 9-hexadecen-1-ol and Erucamide were found, showing promising AChE inhibition through specific interactions [266]. The neuroprotective properties with their mode of action of some plant-associated fungal endophyte-derived bioactive compounds have been given in Table 6.

**Table 6:** Neuroprotective effect and mechanism of action of some of the bioactive compounds derived from fungal endophytes

Medicinal plants	Endophytes	Bioactive compounds	Neuroprotective Activity	Mechanism	Reference
*Houttuynia cordata*	*Penicillium brefeldianum* F4a	Neobrefeldin	AChE and BuChE inhibition activity with IC_50_ values of 0.12 ± 0.05 µM and 175.04 ± 9.16 µM, respectively	It targets both AChE enzyme sites which are PAS and CAS, interacting hydrophobically with Phe295, Phe297, Tyr337, and Phe338 at CAS and forming hydrogen bonds with Tyr124 and hydrophobic interactions with Trp86 at PAS	[267, 268]
*Morus alba*	*Colletotrichum* sp. JS0367	Evariquinone (21)	Neuroprotective effect towards murine hippocampal HT22 cells having an IC_50_ concentration of 42.2 μM	↓ROS, ↓Ca^2+^, ↓MAPKs (JNK, ERK1/2, and p38)	[254]
*Tinospora cordifolia*	*Nigrospora oryzae* (GL15)	Quercetin (22) and (GL15) isolates	Anti‐dementia‐like effect in scopolamine (SCO)-induced mice	↓AChE	[256]
*Salvia przewalskii*	*Alternaria alternata*	Altenusin B, dehydroaltenusin, altenusin, and alterlactone	Neuroprotective effect towards 6-hydroxydopamine (6-OHDA)- or H_2_O_2_-mediated oxidative injury in PC12 cells	↑Nirf-2	[269]
*Euphorbia* sp.	*Fusarium* spp.	OQ-Fus-2-F	Inhibition of the AChE activity having an IC_50_ concentration of 177.0 ± 13.7 µg/mL	↓AChE	[270]
Unidentified	*Fusarium lateritium* SSF2	4,6′-anhydrooxysporidinone	Neuroprotective effect towards glutamate-mediated oxidative injury in HT22 cells	↑Nrf2/HO^−1^ pathways	[271]
*Huperzia serrata*	*Alternaria brassicae* AGF041	AGF041 (Huperzine A)	AChE inhibition effect (75.5 ± 0.7%)	↓AChE	[272]
*Glehnia littoralis*	*Neosartorya fischeri* JS0553	Fischerin (23)	Neuroprotective effect towards glutamate-induced oxidative injury in HT22 cells	↓ROS, ↓Ca^2+^, ↓MAPKs	[255]
*Suaeda japonica*	*Colletotrichum gloeosporioides* JS419	Colletotrichamide C (25)	Neuroprotective effect towards glutamate-induced neurotoxicity in HT22 cells	↓Ca^2+^	[273]
*Psidium littorale*	*Alternaria alternate*	Isosclerone, indole-3-methylethanoate, and ergosta4,6,8(14),22-tetraen-3-one	Potential neuroprotective effect toward glutamate-mediated injury in PC12 cell	↓Ca^2+^	[274]
*Cistanche deserticola*	*Penicillium chrysogenum* No. 005	Chrysogenamide A	Potential neuroprotective effect toward oxidative damaged SH-SY5Y cells	↓ROS	[252]
*Bruguiera gymnorrhiza*	*Penicillium citrinum*	(Z)−7,4′-dimethoxy-6-hydroxy-aurone-4-O-β-glucopyranoside	Potential neuroprotective effect toward 1-methyl-4-phenylpyridinium-mediated oxidatively damaged PC12 cells	Mitochondrial membrane potential (MMP) damage	[253]
*Morus alba*	*Fusarium Solani* JS-0169	Fusarubin (24)	Potential neuroprotective effect toward glutamate-mediated injury in HT22 cells	↓ROS	[275]

↑ upregulation, ↓ downregulation

## Fungal Endophyte-Derived Compounds with Antihypercholesterolemic and Lipid-Lowering Activity

Antihypercholesterolemic drugs are compounds that assist in the reduction of elevated amounts of cholesterol in the bloodstream, thereby treating hypercholesterolemia and lowering the probability of cardiovascular diseases [276]. Lovastatin (26) is a well-known compound that acts as an inhibitor of the HMG-CoA reductase, a crucial enzyme for cholesterol biosynthesis [277, 278]. Several studies demonstrated that cholesterol-lowering compounds were also produced by plants and their associated fungal endophytes [279–281]. The fungal endophyte *Stachybotrys chartarum* that survived in the *Niphates recondita* led to the production of 16 new phenylspirodrimanes known as chartarlactams A–P and eight identified analogues. The stachybotrys chartarum-derived compounds chartarlactams D–F, K–L, N–O, and N-(2-benzenepropanoic acid) stachybotrylactam exhibited potent antihyperlipidemic activity in hepatocarcinoma HepG2 cells at a dose of 10 μM [282]. A fungal endophyte *Diaporthe arengae* TATW2, derived from the host plant *Terminalia arjuna* Roxb., generated three bioactive compounds, namely, benzene propionic acid, 3, 5-bis (1,1-dimethylethyl)−4-hydroxy methyl ester; and pterin-6-carboxylic acid, along with 2,6-ditert-butyl-4-phenol. In vivo, effectiveness experiments of all three isolated chemicals on albino Wistar rats indicated major alterations in the albino Wistar rats’ serum and tissue lipid profiles. *Diaporthe arengae* TATW2 crude extract also exhibited potent anti-hypercholesterolemic action in biochemical test along with reduced lipid peroxidation regarding hRBCs [283]. A fungal endophyte *Diaporthe* sp. JC-J7 dwells inside the stem tissue of the plant *Dendrobium nobile* Lindl. and produces 11 polyketones namely diaporthsins (A to K). The compound diaporthsin E (27), derived from *Diaporthe* sp. JC-J7, showed antihyperlipidemic action towards triglycerides (TG) using steatotic L-02 cells with inhibition ratios of 26% [284]. The fungal endophytes *Trichoderma* sp. CNB 2.5.3, an unidentified fungus CND 2.5.4, along with *Nigrospora* sp. CND 2.1.1, were isolated from the leaf as well as stem tissues of the host plant *Cymbopogon nardus*. These three potent fungal strains produced significant levels of the bioactive compound lovastatin (26). Fungal endophytes have the potential to synthesize lovastatin (26) may be studied in order to optimize fermentation processes to achieve efficient lovastatin (26) synthesis and to investigate its potential in cholesterol-lowering activity [285]. Liu et al. (2024) evaluated the lipid-lowering effect of lovastatin derivatives, specifically seven new compounds: aculeatiol A, aculeatiol B, aculeatiol C, aculeatiol D, aculeatiol E, aculeatiol F, and aculeatiol G, along with known compounds, namely, peniciversiol C, versiol, and decumbenone B, which were isolated from *Aspergillus aculeatus*, a fungal endophyte that survived in the host plant *Morinda citrifolia*. Aculeatiol F structure had unique aromatized heterotetracyclic of ring system with 6/6/6/6 characteristics. The structure of aculeatiol F features a distinctive aromatic heterotetracyclic ring system of 6/6/6/6. Each of these 10 compounds exhibited various levels of lipid-lowering effects against FFA (free fatty acid)-stimulated in HepG2 cells. Remarkably, aculeatiol E demonstrated a notable lipid-lowering effect, including reductions in FFA, oil droplet formation, and intracellular triglycerides, in a dose-dependent manner in HepG2 cells. Aculeatiol E also downregulates the lipid-metabolic–related proteins and enzymes such as FATP2 (fatty acid transport protein 2), FATP5 (fatty acid transport protein 5), FAS (fatty acid synthase), and SCD1 (stearoyl-CoA desaturase 1). Aculeatiol E not only downregulates lipid-metabolism–related proteins and enzymes like FATP2, FATP5, and FAS, and SCD1 but also upregulates the expression of *PPARα* and *ACOX1* genes, indicating its potential as a lipid-lowering agent and for future drug for improving lipid metabolism disorders [286]. Although fungal endophyte–derived potent compounds showed promise in reducing cholesterol levels, further research will be required to completely comprehend their mode of action along with assessing their effectiveness as well as safety for therapeutic use. The antihypercholesterolemic properties of some plant-associated fungal endophyte-derived bioactive compounds have been given in Table 7).

**Table 7:** Antihypercholesterolemic and lipid-lowering compounds derived from fungal endophytes

Medicinal plants	Endophytes	Bioactive compounds	Antihypercholesterolemic activity	Reference
*Siegesbeckia pubescens* Makino	*Colletotrichum capsici*	Collecapsin A and collecapsin B	Both compounds exhibited potential lipid-lowering effect by inhibiting the action of enzyme HMG-Co ~ A reductase having IC_50_ concentrations of 8.72 μM and 15.28 μM, respectively	[287]
*Niphates recondita*	*Stachybotrys chartarum*	Chartarlactams D–F, K–L, N–O, and *N*-(2-benzenepropanoic acid) stachybotrylactam	Potential lipid-lowering effect against hepatocarcinoma cells HepG2 at dose of 10 μM	[282]
*Avicennia marina*	*Aspergillus luchuensis* MERV10	Lovastatin (26)	Antihypercholesterolemic (lowering the low-density lipoprotein cholesterol production)	[278, 288, 289]
*Meretrix meretrix*	*Colletotrichum gloeosporioides* BB4	(10S,11R,13S)-Colletotrichindole A, colletotrichindole B, and ( +)-alternatine A	Potential lipid-lowering effect against 3T3-L1 cells having EC_50_ concentrations of 5.8 μM, 9.0 μM, and 1.3 μM, respectively	[290]
*Cymbopogon nardus* L	*Trichoderma* sp., CNB 2.5.3, an unidentified fungus CND 2.5.4, along with *Nigrospora* sp. CND 2.1.1	Lovastatin (26)	Lipid-lowering effect	[285]
*Taxus baccata*	*Aspergillus niger* PN2	Lovastatin (26)	Potential cytotoxic effect against HeLa and HepG2 cells	[291]
*Piper longum* L	*Periconia* sp.	Piperine (8)	Hypolipidemic effect by internalizing of the cholesterol transporter proteins	[292, 293]
*Terminalia arjuna* Roxb	*Diaporthe arengae* TATW2	Crude extract	Reduction of TC (total cholesterol), TG (triglycerides), and VLDL (very low density lipoproteins) as well as LDL (low-density lipoprotein)	[283]

**Fig. 4:**
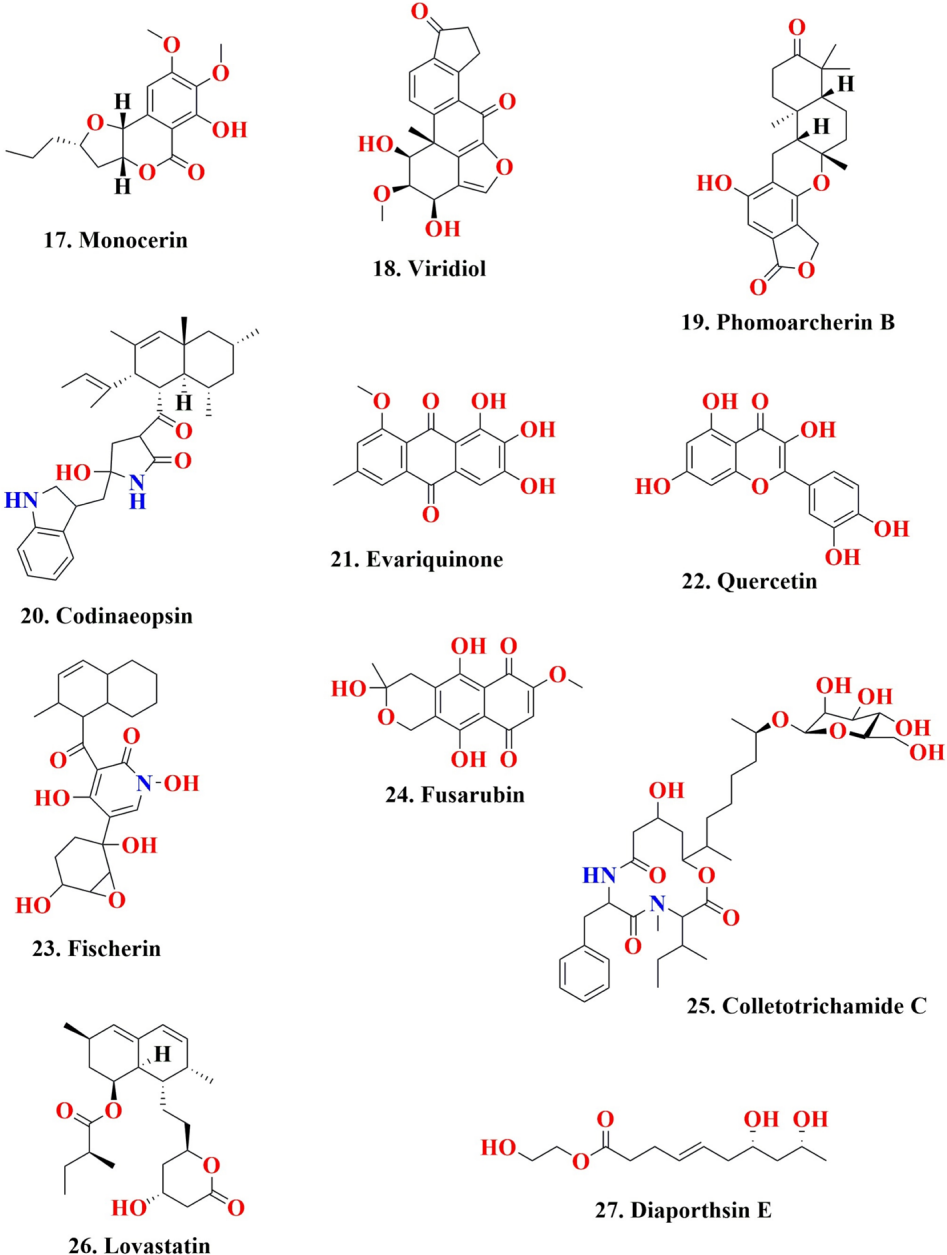
Elucidates the chemical structure of fungal endophytes derived biologically active compounds

In Fig. 3 and Fig. 4, a total of 27 potent metabolites derived from fungal endophytes are mentioned. These compounds have various biological activities such as antimicrobial, antioxidants, anticancer antidiabetic, neuroprotective, and anti-hypercholesterolemic. The compounds mentioned in the above figure are (1) danthron, (2) xanthoascin, (3) fusarithioamide A, (4) phomoenamide, (5) altersolanol A, (6) emodin, (7) epoxydon, (8) piperine, (9) chaetominine, (10) chrysin, (11) epicocconigrone A, (12) scequinadoline D, (13) asperpyridone A, (14) herbarin, (15) neglectine A, (16) epoxycytochalasin H, (17) monocerin, (18) viridiol, (19) phomoarcherin B, (20) codinaeopsin, (21) evariquinone, (22) quercetin, (23) fischerin, (24) fusarubin, (25) colletotrichamide C, (26) lovastatin, and (27) diaporthsin E.

Each compound has peculiar structural characteristics. For example, the emodin is an anthraquinone-derivative compound and exhibits antimicrobial properties. Emodin has polar substituents — hydroxyl group at C6 position, and it was found that this polar functional group can increase antibacterial activity [294]. The fungal endophyte-derived emodin is reported for antimicrobial activities, but their clear mode of action is not reported available so far in my knowledge. The antimicrobial mechanism of action of anthraquinones is bacterial cell wall biosynthesis along with membrane inhibition, DNA synthesis inhibition, several metabolic process inhibitions, etc.

## Endophytic Fungus Biotransform to Produce Potent Derivative Metabolites — a Beneficial Boon

**Fig. 5:**
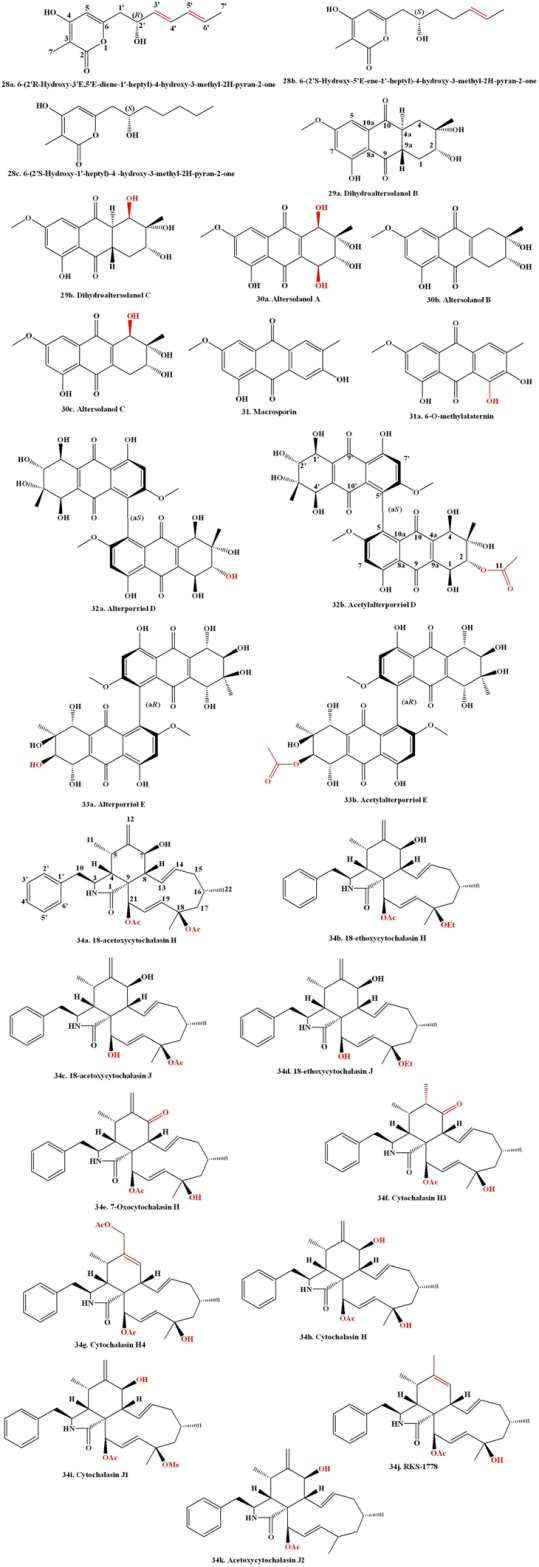
Elucidates the chemical structures of compounds from fungal endophytes for exploring the structure-bioactivity relationships

**Fig. 6:**
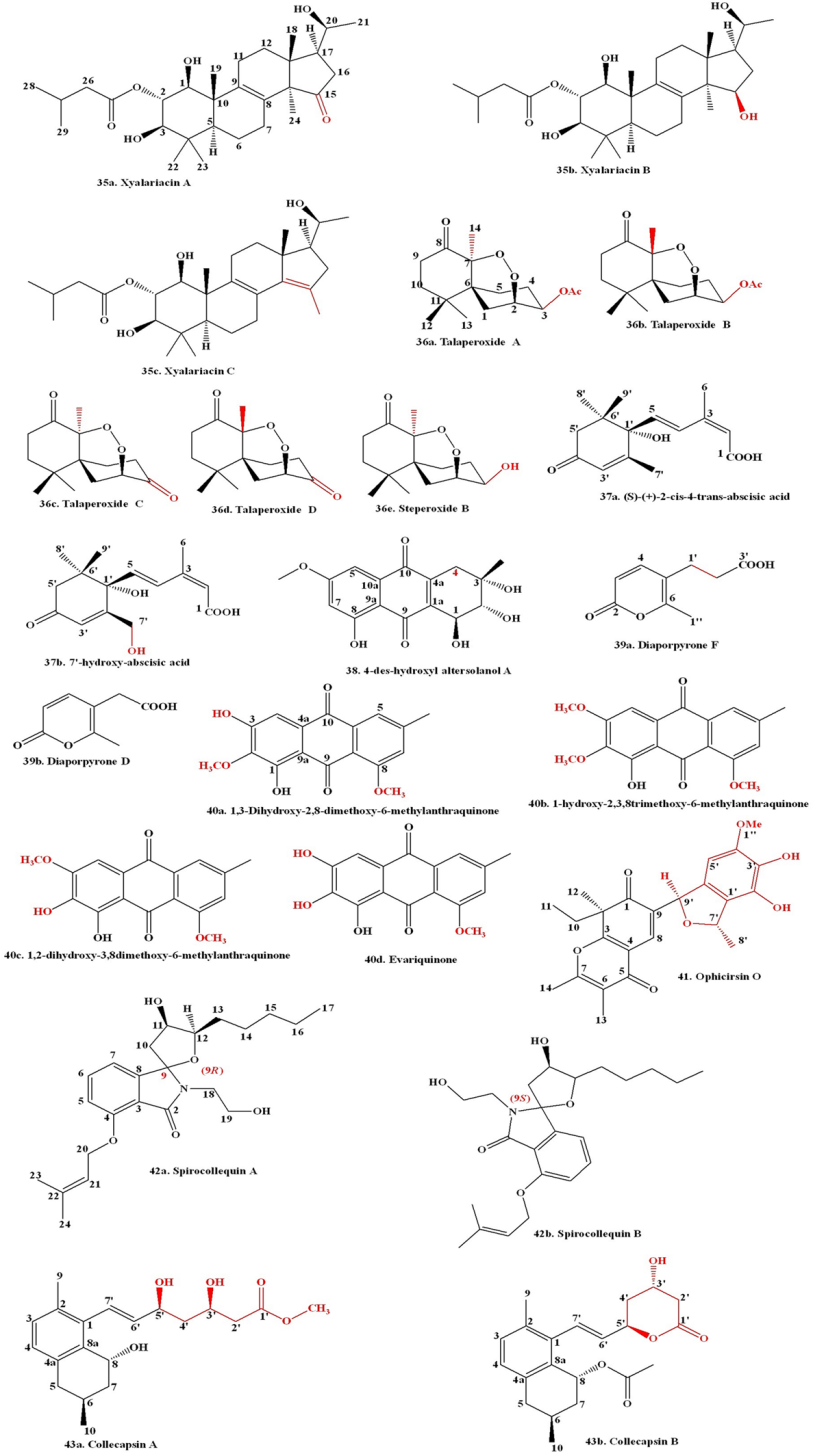
Elucidates the chemical structures of compounds from fungal endophytes for exploring the structure-bioactivity relationships

Fungal endophytes could transform both simple and complicated substrates into new molecules via a variety of chemicals along with enzymatic reactions that occur within their cells [295–299]. The fungi endophytes use a variety of chemical processes through their biochemical pathways, including hydrolysis, condensation, cyclization, reduction, and oxidation to produce secondary metabolites [300–307]. Additionally, a variety of enzymes that catalyze specific biochemical reactions are present in fungi endophytes, including hydrolases, isomerases, oxidoreductases, transferases, lyases, and ligases [308–314]. Overall, these chemical and enzymatic processes in fungal endophytes could alter precursor molecules, resulting in structurally diverse derivative products [315–318]. Fungal endophyte-mediated transformation has been widely utilized to create an immense quantity of novel metabolites, increasing specificity along with bioactivity [319–323]. Fungal endophytes create robust derivative compounds with additional functional groups, which could enhance their effectiveness by allowing for specific interactions with biological systems (Fig. 5 and Fig. 6) [97, 296, 324–326]. α-Pyrone represents a heterocyclic molecule that is categorized as a lactone with abundance in bioactive metabolites. It has a functional component called an alpha ketone attached to its pyran ring. The compounds 6-(2′R-hydroxy-3′E,5′E-diene-1′-heptyl)−4-hydroxy-3-methyl-2H-pyran-2-one (28a) and 6-(2′S-hydroxy-5′E-ene-1′-heptyl)−4-hydroxy-3-methyl-2H-pyran-2-one (28b), as well as 6-(2′S-hydroxy-1′-heptyl)−4 -hydroxy-3-methyl-2H-pyran-2-one (28c), were alpha-pyrone derivatives obtained from a fungal endophyte *Penicillium ochrochloronthe* and survive in the *Taxus media* plant. The antifungal activities of these three compounds towards the tested 20 pathogenic fungal strains with MIC values were as follows: *Cercospora arachidicola* Hori (12.5 µg/ml, 12.5 µg/ml, and 25 µg/ml), *Alternaria solani* (12.5 µg/ml, 25 µg/ml, 12.5 µg/ml), *Bipolaris carbonum* Wilson (25 µg/ml, 25 µg/ml, 25 µg/ml), *Fusarium graminearum* (12.5 µg/ml, 12.5 µg/ml, 50 µg/ml), *Cylindrocladium parasiticum* (25 µg/ml, 25 µg/ml, 12.5 µg/ml), *Alternaria alternata f.* sp. *mali* (25 µg/ml, 50 µg/ml, 12.5 µg/ml), *Cercospora personata* (25 µg/ml, 25 µg/ml, 50 µg/ml), *Botrytis cinerea* Pers. (25 µg/ml, 50 µg/ml, 25 µg/ml), *Ustilago scitaminea* Syd. (25 µg/ml, 25 µg/ml, 50 µg/ml), *Rhizoctonia cerealis* (50 µg/ml, 50 µg/ml, 25 µg/ml), *Helminthosporium maydis* (50 µg/ml, 50 µg/ml, 50 µg/ml), *Colletotrichum orbiculare* (50 µg/ml, 50 µg/ml, 50 µg/ml), *Ascochyta gossypii* Syd. (50 µg/ml, 50 µg/ml, 50 µg/ml), *Alteraria alternata (Fries) Keissler* (50 µg/ml, 50 µg/ml, 50 µg/ml), *Colletortrichum gloeosporiodes* (25 µg/ml, 100 µg/ml, 50 µg/ml), *Colletotrichum graminicola* (100 µg/ml, 25 µg/ml, 25 µg/ml), *Botrytis fabiopsis* (50 µg/ml, 50 µg/ml, 100 µg/ml), *Alternaria brassicae* (50 µg/ml, > 100 µg/ml, > 100 µg/ml), *Sclerotinia sclerotiorum* (25 µg/ml, 100 µg/ml, > 100 µg/ml), *Exserohilum turcicum* (100 µg/ml, > 100 µg/ml, > 100 µg/ml), respectively. However, these three compounds demonstrated antibacterial activity against the following tested pathogenic bacteria with MIC values: *Bacillus subtilis* (50 µg/ml, 50 µg/ml, 50 µg/ml), *Micrococcus luteus* (50 µg/ml, 50 µg/ml, 50 µg/ml), *Staphylococcus aureus* (50 µg/ml, 50 µg/ml, 50 µg/ml), *Bacillus megaterium* (50 µg/ml, 50 µg/ml, 50 µg/ml), *Salmonella enterica* (50 µg/ml, 50 µg/ml, 50 µg/ml), *Proteusbacillus vulgaris* (50 µg/ml, 50 µg/ml, 50 µg/ml), *Salmonella typhi* (50 µg/ml, 25 µg/ml, 50 µg/ml), *Pseudomonas aeruginosa* (50 µg/ml, 50 µg/ml, 50 µg/ml), *Escherichia coli* (50 µg/ml, 50 µg/ml, 50 µg/ml), and *Enterobacter aerogenes* (50 µg/ml, 100 µg/ml, 50 µg/ml), respectively. Structurally, the compounds 6-(2′R-hydroxy-3′E,5′E-diene-1′-heptyl)−4-hydroxy-3-methyl-2*H*-pyran-2-one (28a), 6-(2′S-hydroxy-5′E-ene-1′-heptyl)−4-hydroxy-3-methyl-2H-pyran-2-one (28b), and 6-(2′S-hydroxy-1′-heptyl)−4-hydroxy-3-methyl-2*H*-pyran-2-one (28c) were alpha-pyrone derivatives. The sole difference between them lies in the unsaturation present within the hydrocarbon chain. Consequently, both their antifungal and antibacterial activities against certain species were almost comparable. According to this, their bioactivities of derivative compounds are not affected much by the slight variation that occurs in the hydrocarbon chain’s process known as dehydrogenation [327]. Eleven compounds, including dihydroaltersolanol B (29a), dihydroaltersolanol C (29b), acetylalterporriol D (32b), acetylalterporriol E (33b), altersolanol A (30a), altersolanol B (30b), altersolanol C (30c), alterporriol D (32a), alterporriol E (33a), macrosporin (31), and 6-O-methylalaternin (31a), were derived from *Stemphylium globuliferum*, a fungal endophyte dwelling in the host plant *Juncus acutus*. Structurally, compounds dihydroaltersolanol B (29a) and dihydroaltersolanol C (29b) were dihydro derivatives of altersolanol B (30b) and altersolanol C (30c), respectively. Dihydroaltersolanol C (29b) was structurally identical to dihydroaltersolanol B (29a), differing only by an extra hydroxy group located in the C-4 position. Remarkably, only dihydroaltersolanol C (29b) exhibited modest antibacterial activity towards *Staphylococcus aureus* ATCC 29213 among the four compounds (29a, 29b, 32b, and 33b) evaluated, with a MIC concentration of 49.7 μM. Dihydroaltersolanol C (29b) also exhibited significant cytotoxicity towards L5178Y cells having IC_50_ concentration of 3.4 μM. This suggests that the additional hydroxy group at the C-4 position of dihydroaltersolanol C (29b) contributes to its enhanced bioactivities. Three derivatives of altersolanol, namely, altersolanol A-C (30a, 30b, and 30c), showed potent cytotoxicity towards L5178Y cells, having IC_50_ concentrations of 2.53 μM, 3.78 μM, and 4.68 μM, respectively. This suggests that substituents on the aliphatic cycle have minimal potential to alter bioactivity efficacy. Moreover, the derivative of anthraquinone compound 6-O-methylalaternin (31a) along with its analogue macrosporin (31) was tested for their cytotoxicity potential. Interestingly, 6-O-methylalaternin (31a) showed significant percent growth inhibition (98.1%) of L5178Y cells at a particular concentration represent 10 µg/ml along with IC_50_ concentration of 1.25 μM. On the other hand, macrosporin (31) showed low cytotoxic activity on L5178Y cells with 45.5% growth inhibited at similar concentration representing 10 µg/ml. This highlights the importance of ortho-dihydroxy substitution in enhancing the compound’s cytotoxic efficacy. Surprisingly, acetylalterporriol E (33b) and alterporriol E (33a), both possessing axial chirality of (aR), demonstrated potent cytotoxic activity towards L5178Y cells, having IC_50_ concentrations of 10.4 μM and 6.9 μM, respectively. Conversely, their (aS) counterparts showed no activity. The findings suggest that the potent derivative chemicals and related congeners possess various modes of action [328]. The fundamental structure of intricate natural chemicals called cytochalasans is composed of a macrocyclic ring joined with a perhydroisoindolone ring. Together with polyketide-derived additional chains and distinct functional groups, as well as their fundamental stereochemical elements, play important roles in an array of biological activities, especially in anticancer actions. Eleven derivative compounds of cytochalasans were isolated from an endophytic fungal strain, *Phomopsis* sp. shj2, residing within the stem tissue of the host plant *Isodon eriocalyx* var. *laxiflora*. These compounds include 18-acetoxycytochalasin H (34a), 18-ethoxycytochalasin H (34b), 18-acetoxycytochalasin J (34c), 18-ethoxycytochalasin J (34d), 7-oxocytochalasin H (34e), cytochalasin H_3_ (34f), cytochalasin H_4_ (34 g), cytochalasin H (34 h), cytochalasin J_1_ (34i), RKS-1778 (34j), and acetoxycytochalasin J_2_ (34 k). The compounds 18-acetoxycytochalasin H, 18-ethoxycytochalasin H, 18-acetoxycytochalasin J, cytochalasin H, cytochalasin J_1_ (34i), RKS-1778 (34j), and acetoxycytochalasin J_2_ (34 k) showed considerable antimigratory property towards MDA-MB-231 cells having IC_50_ concentrations of 3.14 μM, 10.42 μM, 6.38 μM, 1.25 μM, 7.31 μM, 1.01 μM, and 6.41 μM, respectively. The reduced anti-migratory activity of 18-acetoxycytochalasin H (34a) and 18-acetoxycytochalasin J (34c), along with cytochalasin J_1_ (34i), compared to cytochalasin H (34 h), was attributed to substituting an acetoxy, ethoxy, and methoxy functional group for the C-18 hydroxy functional group. Acetoxycytochalasin J_2_ (34 k) had higher anti-migratory action than both 18-Ethoxycytochalasin H (34b) and cytochalasin J_1_ (34i), which might be attributed to an extra dual bond among C-17 and C-18. The better anti-migratory action of 18-acetoxycytochalasin H (34a) over 18-acetoxycytochalasin J (34c) seems probably due to the inclusion of an extra acetoxy group located at the C-21 position. When comparing the structure of cytochalasin H_4_ (34 g), cytochalasin H (34 h), and RKS-1778 (34j), substituting double bonds and hydroxy with tri-substituted alkene increased activity while adding acetoxy decreased it. As a result, this structure with activity interaction investigation might aid in the biosynthesis of new anticancer compounds [329]. Three new triterpene derivative compounds such as xylariacin A (35a), xylariacin B (35b), and xylariacin C (35c) were derived from an endophytic fungal strain *Xylarialean* sp. A45, which was isolated from the plant *Annona squamosa* L. Xylariacins A–C (35a, 35b, and 35c) exhibited cytotoxic effects against HepG2 cells, with percent growth inhibition values of 48.0%, 9.7%, and 46.7%, respectively, at a specific value of 20 µg/ml. The structural variance between xylariacin A (35a) and xylariacin B (35b) involves the substitution of a hydroxy group with an oxy group at the C-15 position. This substitution could potentially impact the bioactivity potential of both compounds [330]. The compounds talaperoxides A to D (36a, 36b, 36c, and 36d) and steperoxide B (36e) were norsesquiterpene peroxide derivatives obtained from *Talaromyces flavus*, a fungal endophyte that survives in the host plant *Sonneratia apetala*. Talaperoxide A (36a) shared a similar skeletal structure with talaperoxide B (36b), differing only in the orientation at the C-7 position, indicating an epimeric relationship resulting from this alteration. Similarly, talaperoxide D (36d) acts as epimer of talaperoxide C (36c) differing only in the orientation at the C-7 position. Steperoxide B (36e) also shared a common skeletal structure with talaperoxide A (36a), differing only in the substitution of an acetoxy group with a hydroxy group at the C-3 position. The cytotoxic effects of these compounds against various cell lines, including MCF-7 cells, MDA-MB-435 cells, HepG2 cells, and HeLa cells, as well as PC-3 cells, exhibited IC_50_ concentrations that ranged as follows: talaperoxide A (36a) (5.70 to 19.77 µg/ml), talaperoxide B (36b) (0.89 to 2.78 µg/ml), talaperoxide C (36c) (2.64 to 15.11 µg/ml), talaperoxide D (36d) (0.70 to 1.92 µg/ml), and steperoxide B (36e) (1.82 to 7.97 µg/ml), respectively. The study clearly indicates that alterations in chemical structure, resulting from different orientations and functional groups, significantly affect the efficacy of bioactive compounds [331]. Two derivatives of abscisic acid, namely (S)-( +)−2-cis-4-trans-abscisic acid (37a) and 7′-hydroxy-abscisic acid (37b), along with a derivative of altersolanol A known as 4-des-hydroxyl altersolanol A (38), were derived from a fungal endophyte, *Nigrospora oryzae*, which survived in the leaf tissue of the host plant *Combretum dolichopetalum*. (S)-( +)−2-cis-4-trans-abscisic acid (37a) and 7′-hydroxy-abscisic acid (37b) share a similar core structure. The main distinction was the extra hydroxy group at position C-7′ in 7′-hydroxy-abscisic acid (37b). The compounds (S)-( +)−2-cis-4-trans-abscisic acid (37a), 7′-hydroxy-abscisic acid (37b), and 4-des-hydroxyl altersolanol A (38) showed antidiabetic effects, lowering blood sugar levels by 5.92%, 44.96%, and 43.70%, respectively, in alloxan-stimulated diabetic mice over a 9-h treatment, as measured by fasting blood sugar levels. Thus, an additional hydroxy group of 7′-hydroxy-abscisic acid (37b) could increase their antidiabetic potential [332]. Recently, diaporpyrone F (39a) and diaporpyrone D (39b), both pyrone derivatives, were extracted from an endophytic fungal strain known as *Diaporthe* sp. (CB10100), which dwells in the *Sinomenium acutum* plant. Both compounds share a similar skeletal structure, differing only in the presence of an ethanoic group in diaporpyrone D (39b) and a propanoic group in diaporpyrone F (39a), both located at the C-5 position of the core pyrone ring. Remarkably, diaporpyrone D (39b) showed a 46.40% inhibition of α-glucosidase activity at a concentration of 800 μM, whereas diaporpyrone F (39a) demonstrated no such inhibition at the same concentration. This suggests that the carbon chain length of the functional group may influence the antidiabetic effectiveness of pyrone-derived compounds [333]. Another structure–activity study revealed that four anthraquinone derivative compounds, namely, 1,3-dihydroxy-2,8-dimethoxy-6-methylanthraquinone (40a), 1-hydroxy-2,3,8trimethoxy-6-methylanthraquinone (40b), 1,2-dihydroxy-3,8dimethoxy-6-methylanthraquinone (40c), and evariquinone (40d) were isolated from an endophytic fungal strain *Colletotrichum* sp. (JS-0367), which survives in the host plant *Morus alba*. The fundamental core structure of all four compounds remained similar, with differences observed in the functional groups positioned at various locations on the anthraquinone ring. Evariquinone (40d) had a distinctive hydroxy group at position of C-1, C-2, and C-3 of the anthraquinone ring. Structurally, the compounds 1,3-dihydroxy-2,8-dimethoxy-6-methylanthraquinone (40a), 1-hydroxy-2,3,8trimethoxy-6-methylanthraquinone (40b), and 1,2-dihydroxy-3,8dimethoxy-6-methylanthraquinone (40c) had substitution of methoxy group with hydroxy group at position of C-2 for 1,3-dihydroxy-2,8-dimethoxy-6-methylanthraquinone (40a), and at position of C-2 and C-3 for 1-hydroxy-2,3,8trimethoxy-6-methylanthraquinone (40b) and C-3 position for 1,2-dihydroxy-3,8dimethoxy-6-methylanthraquinone (40c) in comparison with evariquinone (40d). Remarkably, evariquinone (40d) exhibited greater neuroprotective activity towards glutamate-mediated oxidative damage in HT22 cells in comparison with other three anthraquinone derivative compounds. Evariquinone (40d) also demonstrated antioxidant characteristics towards DPPH-free radicals with IC_50_ concentration of 42.2 μM. The enhancement in the neuroprotective activity of these anthraquinones correlates with increased hydroxyl substitution, trapping excess electrons along with balancing ROS levels, while methylation of hydroxyl groups may reduce neuroprotection [254]. Sixteen polyketide derivatives namely ophicirsins A to P were derived from a fungal endophyte *Ophiobolus cirsii* (LZU-1509), which was obtained from the plant *Anaphalis lactea*. None of the 16 compounds had cytotoxic activities against the tested cell lines such as HepG2 and HT-1080 cells at a concentration of 20 µM. However, among these compounds, only ophicirsin O (41) exhibited significant neuroprotection against H_2_O_2_-induced neurotoxicity in PC-12 cells, along with strong antioxidant activity against DPPH-free radicals, surpassing the effectiveness of resveratrol, the standard drug. Ophicirsin O (41) represents a novel polyketide structure, comprising a hydrogenated benzopyran fused with a benzofuran. Ophicirsin O (41) contains hydroxy groups at C-2′ and C-3′, which could potentially contribute to its neuroprotective action by readily transferring electrons to scavenge free radicals [334]. Two diastereomeric isoindolinone alkaloid compounds, spirocollequin A (42a) and spirocollequin B (42b), originated from an endophytic fungal strain *Colletotrichum boninense* (AM-12–2). Both compounds exhibit a core structure characterized by 4,5-dihydro-spiro[furan-2,1′-isoindolin]−3′-one. Spirocollequin A (42a) and spirocollequin B (42b) showed antiplasmodial activity towards *Plasmodium falciparum* (3D7) having IC_50_ concentration of 9.72 ± 0.61 µM and 4.71 ± 0.77 µM along with cytotoxicity against HFF (human foreskin fibroblast) cells with IC_50_ concentrations of 246.8 ± 10.5 µM and 266.2 ± 3.1 µM, respectively. Spirocollequin A (42a) possesses a configuration of 9R11R12R, while spirocollequin B (42b) features a configuration of 9S11R12R. The variance in (R) and (S) configurations at C-9 could potentially enhance the antimalarial potency while also promoting safer characteristics for spirocollequin B (42b) [335]. Two undescribed polyketide derivatives, namely, collecapsin A (43a) and collecapsin B (43b), together with two previously described compounds macrolactin A and macrolactin B, were derived from a fungal endophyte *Colletotrichum capsici*, which was isolated from the plant *Siegesbeckia pubescens* Makino. Collecapsin A (43a) and collecapsin B (43b) exhibit distinct skeleton structures. Collecapsin A (43a) is characterized by a methyl ester chain with hydroxy groups at C-3′ and C-5′ positions, while collecapsin B (43b) showcases a tetrahydropyron ring attached at the C-5′ with a keto group at C-1′ along with a hydroxy group at C-3′ position. Furthermore, collecapsin B (43b) diverges from collecapsin A (43a) which appeared with a substitution of the hydroxy group at the C-8 position by a carboxy group. Remarkably, both compounds collecapsin A (43a) and collecapsin B (43b) exhibited considerable inhibition of HMG-Co ~ A reductase activity having IC_50_ concentrations of 8.72 μM and 15.28 μM, respectively. The reduced activity of collecapsin B (43b) could result from steric hindrance caused by the bulkier group and the substitution of the hydroxy group with a carboxy group at the C-8 position [287]. Thus, fungi endophytes are now utilized to biotransform potent bioactive compounds with its efficacious derivatives [296, 336–340]. Fungal endophytes produce diverse metabolites but lack comprehensive pharmacological understanding and structure–activity relationship data [341–345]. This study could reveal the efficacy of derivative metabolites and create opportunities to synthesize targeted potent derivatives with high bioactivity potential.

Fungal endophytes convert substrates into novel molecules through diverse chemical processes, producing secondary metabolites with various enzymatic activities. For example, alpha-pyrone derivatives like 6-(2′R-hydroxy-3′E,5′E-diene-1′-heptyl)−4-hydroxy-3-methyl-2*H*-pyran-2-one exhibit antifungal and antibacterial properties, while *Stemphylium globuliferum* metabolites, including dihydroaltersolanol B and C, show cytotoxicity and antibacterial activity influenced by structural modifications. Cytochalasans from *Phomopsis* sp. possess antimigratory properties against cancer cells, and Xylariacins from *Xylarialean* sp. exhibit cytotoxic effects on HepG2 cells, with bioactivity affected by functional group variations. Norsesquiterpene peroxides, like talaperoxides and steperoxide B, display cytotoxicity against cancer cell lines, and abscisic acid derivatives, such as (S)-( +)−2-cis-4-trans-abscisic acid, show antidiabetic effects enhanced by additional hydroxy groups. Pyrone derivatives, including diaporpyrone D and F, influence α-glucosidase inhibition based on carbon chain length, while anthraquinone derivatives like evariquinone demonstrate neuroprotective and antioxidant activities, with increased hydroxyl substitution correlating with enhanced effects. Polyketide derivatives from *Ophiobolus cirsii* show neuroprotective properties without cytotoxicity, underscoring the impact of structural variations on bioactivity potential.

Figure 5 and Figure 6 represent a total of 47 derivative metabolites derived from fungal endophytes. These compounds have various biological activities such as antimicrobial, antioxidants, anticancer antidiabetic, neuroprotective, and anti-hypercholesterolemic. The compounds mentioned in the above figure are 6-(2′R-hydroxy-3′E,5′E-diene-1′-heptyl)−4-hydroxy-3-methyl-2*H*-pyran-2-one (28a), 6-(2′S-hydroxy-5′E-ene-1′-heptyl)−4-hydroxy-3-methyl-2*H*-pyran-2-one (28b), 6-(2′S-hydroxy-1′-heptyl)−4 -hydroxy-3-methyl-2*H*-pyran-2-one (28c), dihydroaltersolanol B (29a), dihydroaltersolanol C (29b), altersolanol A (30a), altersolanol B (30b), altersolanol C (30c), macrosporin (31), 6-O-methylalaternin (31a), alterporriol D (32a), acetylalterporriol D (32b), alterporriol E (33a), acetylalterporriol E (33b), 18-acetoxycytochalasin H (34a), 18-ethoxycytochalasin H (34b), 18-acetoxycytochalasin J (34c), 18-ethoxycytochalasin J (34d), 7-oxocytochalasin H (34e), cytochalasin H_3_ (34f), cytochalasin H_4_ (34 g), cytochalasin H (34 h), cytochalasin J_1_ (34i), RKS-1778 (34j), acetoxycytochalasin J_2_ (34 k), xylariacin A (35a), xylariacin B (35b), xylariacin C (35c), talaperoxide A (36a), talaperoxide B (36b), talaperoxide C (36c), talaperoxide D (36d), steperoxide B (36e), (S)-( +)−2-cis-4-trans-abscisic acid (37a), 7′-hydroxy-abscisic acid (37b), 4-des-hydroxyl altersolanol A (38), diaporpyrone F (39a), diaporpyrone D (39b), 1,3-dihydroxy-2,8-dimethoxy-6-methylanthraquinone (40a), 1-hydroxy-2,3,8trimethoxy-6-methylanthraquinone (40b), 1,2-dihydroxy-3,8dimethoxy-6-methylanthraquinone (40c), evariquinone (40d), ophicirsin O (41), spirocollequin A (42a), spirocollequin B (42b), collecapsin A (43a), and collecapsin B (43b).

## Advancement in Approaches to Enhance the Production of Metabolite Associated with Fungal Endophytes

**Fig. 7:**
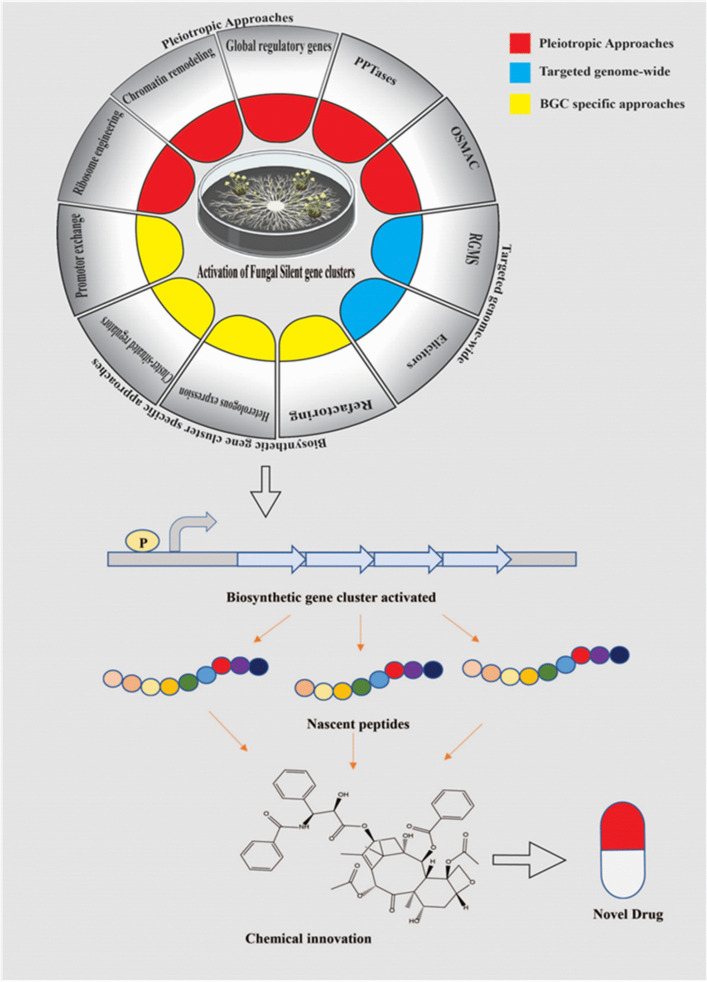
Represents several approaches through activation of cryptic Biosynthetic gene clusters (BGCs) in fungi for enhanced production of fungus derived bioactive compounds [399]. This figure elucidates various approaches to activate cryptic biosynthetic gene clusters of fungi for enhance metabolites production. There are three major approaches i.e., activation of cryptic biosynthetic gene clusters (BGCs) approach, pleiotropic approaches, and targeted genome-wide approaches. In BGCs approach the fungi isolated from the same host can activate the gene cluster by exchanging the silent gene cluster's promoters with constitutively active promoter. Identify and employ Cluster-Specific Transcription Factors (CS-TFs) or Cluster Activation Proteins (CAPs) to regulate the biosynthetic gene clusters. Upregulation of regulatory genes within the cluster may inhibit the repressor and activate the biosynthetic gene clusters, resulting in enhance metabolite production. Heterologous expression involves insertion of the biosynthetic gene cluster to a different host organism with better growth conditions and greater ease of manipulation. Host can be either bacteria or a different fungal strain. Refactoring a dormant gene cluster requires replacing its original promoters with potent or constitutive promoters that promote gene expression, usually with the goal of increasing the production of desirable products. This technique includes identifying the cluster, creating, and modifying new promoters, testing with different expression levels, and analyzing the resultant product. Pleiotropic approaches for enhance metabolic productions utilizes various biotechnological methods such as ribosome engineering, chromatin remodeling, global regulatory genes, PPtases (Phosphopantetheinyl Transferases), OSMAC (One Strain Many Compounds), etc. Mutations in ribosomal proteins, which confer resistance to antibiotics that target the ribosome, have also been observed to trigger the activation of silent genes. The co-regulation of histone acetylation and methylation impacts the transcription of gene clusters responsible for secondary metabolism, commonly situated in sub-telomeric regions. Global regulators control numerous essential gene clusters, and modifying these regulatory networks has the potential to activate biosynthetic gene clusters (BGCs). PPTases play a vital role in modifying carrier proteins after translation, which is a crucial step in the production of polyketides and non-ribosomal peptides. When the native PPTase is downregulated, overexpressing PPTases has the potential to affect the production of secondary metabolites. In the OSMAC approach, fungi are co-cultivated with other fungi or bacteria under various culture conditions. This helps uncover the environmental signals necessary to activate the biosynthesis of secondary metabolites. In targeted genome wide approach employing Reporter Guided Mutant Selection to target pathway promoters which indicate elevated transcription from the BGC. In high-throughput elicitor screening, gene clusters are activated through the discovery of small-molecular weight compounds that stimulate expression. Abbreviations: OSMAC (One Strain Many Compounds); P (Promoters); PPtases (Phosphopantetheinyl Transferases); RGMS (Reporter-Guided Mutant Selection)

The fungal endophyte-derived metabolites with immense structural diversity; varied pharmacological properties, safety, and intrinsic ability to bind to other biological molecules of natural products; and their biocompatibility make it successful in drug discovery [346–349]. There are many studies on the biosynthesis of bioactive compounds of plant-associated fungal endophytes. Consequently the development in fermentation, extraction, purification, and characterization processes as well as bioassay approaches allowed researchers to quickly identify and characterize the potent bioactive compounds of fungal endophytes [105, 350, 351]. Endophytes can bio-transform the original biological molecules derived from naturally occurring phytochemicals into their more potent derivatives, leading to both functionality and structural variation [155, 319, 352, 353]. Various approaches comprising OSMAC, co-culture, epigenetic modifiers, pleiotropic regulators, elicitors, and molecular techniques are utilized for the activation of cryptic biosynthetic gene clusters (BGCs) in endophytic along with non-endophytic fungi to increase secondary metabolite production (Fig. 7) [354–362]. Other molecular-based techniques for the stimulation of cryptic BGCs of fungal endophytes have been extensively studied previously, such as pleiotropic strategies, regulation of global transcription factors, and ribosome, as well as heterologous host transfer [359, 363–368]. Using biotechnological methods for altering the genome of fungal endophytes holds the promise to enhance the production of secondary metabolites along with diversifying the possible uses of these beneficial substances [369–373]. Recent research has shown that integrating modern wide-genome approaches, including genomics, comparative genomics, gene-editing techniques, transcriptomics, and metabolomics, has opened up new pathways for improving secondary metabolite synthesis in fungi. These approaches provide an in-depth awareness of the genetic mechanisms along with regulatory mechanisms underlying secondary metabolite biosynthesis, leading to the identification of novel bioactive chemicals as well as improvements of fungal strains for several applications including the discovery of drugs, agriculture, and industrial biotechnology [370, 374–380]. Metabolomics approaches for fungal endophyte-derived compounds are being used to explore the biotransformation process, the effect of numerous environmental conditions on endophyte metabolome, and the significance of different cultivation procedures using an array of methods including HPLC, HPTLC, LC-HR-MS, GC–MS, FTIR, 1D/2D NMR, and others [381–386]. It is important to note that the selection for each strategies outlined here should be determined by the needs of respective researchers, their degree of experience, and the type of fungal strains, among other variables. Each of these strategies has its own advantages and disadvantages.

## Metabolomics and Multivariate Analysis of Fungal Endophyte-Derived Metabolites

Metabolomics, as a phenotyping tool, represents one of the most effective bioanalytical methods for the discovery and determination of the array of metabolites of biological samples [387–390]. Researchers have encountered difficulties due to complexities in data processing for metabolomics studies. The use of multivariate analysis has an important role in identifying vital metabolite data using huge raw datasets. Principal component analysis (PCA) and hierarchical cluster analysis (HCA), as well as orthogonal partial least squares discriminant analysis (OPLS-DA) methods, are the most extensively utilized for multivariate analysis in metabolomics studies [225, 388, 391–393]. In metabolomic studies, heatmaps are created, which act as efficient preliminary and analytical tools, to uncover the significant patterns, abundance, and distribution of potent metabolites. Thus, it allows exploring the complex metabolomics information, categorizing samples or their byproducts according to expression patterns, determining coordinated alterations, and probable biomarkers related to particular types or conditions [377, 394, 395]. The LC-HR-MS-dependent metabolomics are used to assess the antioxidant characteristics of fungi endophytes obtained from different parts of *Artemisia annua* as well as *Medicago sativa* plants. They employed tools like a heat map and multivariate analysis [396]. The fungal endophyte isolation from various tissues like the leaf, root, and stem of the plant was performed by using malt extract agar medium supplemented with ampicillin to inhibit bacterial growth. The fungal species was identified by their morphological along with molecular characteristics. The fermentation process was performed with liquid malt extract agar medium and the solvent ethyl acetate utilized to extract the fungal crude metabolites. Metabolomic profiling of fungal crude metabolites was accomplished with the LC-HR-MS methods, and multivariate analysis was performed using PCA and OPLS-DA methods to identify the variation between the compositions of fungal metabolites. The biochemical antioxidant assay was performed using free radical scavenging assay, FRAP assay, MDA assay, and xanthine oxidase inhibition assay. Three endophytic fungal strains from *Aspergillus terreus* (AFL, AFSt and AFR) exhibited significant antioxidant potential, and their metabolomic profile showed several bioactive compounds with various classes including phenolic, coumarin, alkaloid, and polyketide [396]. An endophytic fungus *Diaporthe fraxini* ED2 cultured in various mediums exhibited significant antioxidant potential, and LC-HR-MS-based metabolite profile showed several potent metabolites [397]. The fungal endophytes associated with plant *Artemesia annua* showed antimalarial activity. The LC-HR-MS-based metabolite profile of the fungal crude extract and their multivariate analysis revealed that eight bioactive compounds were present. The in vitro study of compounds rmodin (6) and physcion exhibited a potent antimalarial effect having IC_50_ concentrations of 0.9 and 1.9 µM, respectively [225]. *Talaromyces trachyspermus*, a fungal endophytic species, produces several bioactive compounds with various pharmacological properties like antimicrobial, antioxidant, and nematicidal. The method of GC–MS was used to identify all of these bioactive chemicals [398].

Fungal endophyte-derived metabolites showcase immense structural diversity and varied pharmacological properties, making them valuable in drug discovery. Advances in fermentation, extraction, and characterization have accelerated the identification of potent bioactive compounds. Techniques like OSMAC, co-culture, and gene-editing, coupled with metabolomics and multivariate analysis, enhance secondary metabolite production and characterization. Metabolomics, including LC-HR-MS and multivariate analysis, reveals antioxidant, antimalarial, and antimicrobial potentials of endophytes, offering insights into bioactive compound distribution and efficacy.

## Conclusion and Future Prospect

Plants are rich sources of diverse bioactive metabolites which exert various biological functions. This has renewed considerable interest in the quest for new bioactive potent chemicals found in nature, leading to a growing demand for research along with the development of new pharmaceutical drugs in the industry. Even though several medications have been found, screening procedures are still widely used for uncovering new bioactive compounds from natural sources. The potent metabolites of fungal endophytes linked with medicinal plants have received little attention in terms of bioactivity. Because only a few fungal endophytes have been investigated in the available medicinal plants, investigators are concentrating their efforts on evaluating the prospective secondary metabolites generated by these fungi. Fungal endophytes enhance host plant performance during abiotic and biotic stresses by altering factors that influence the plants’ responses. The recent progress in biotechnology and bioinformatics tools, including the CRISPR-Cas system, metabolomics, proteomics, and genomics, has opened the opportunity for molecular level exploration of endophytes. Fungal endophytes have the potential to boost metabolite production via techniques such as genetic manipulation, optimizing growth conditions, co-cultivation, alteration in metabolomic process, elicitation, improving fermentation processes, and employing omics methodologies, to determine potent metabolite insight through plant host-endophyte interactions. Bioprospecting of fungal endophyte on a global scale holds the promise of conserving plant biodiversity and acts as eco-friendliness in ecosystems. Thus, endophytic fungi represents an active reservoir of natural potent bioactive chemicals that are unique, renewable, and relatively low in toxicity, as well as more effective, potent, cheap, and safer, along with less resistance compared to chemically synthesized therapeutic drugs. This would therefore reduce the enormous load upon public healthcare facilities and help the healthcare and pharmaceutical sectors. A number of bioactive compounds and their derivatives generated by fungal endophytes are still to be explored in relation to their host-plant associations, especially their bioactivities, signaling pathways, and mode of action. This gap in research could potentially expose new therapeutic compounds with high potency. Plant-associated endophytes form a crucial symbiotic relationship with plants, acting as bio-reservoirs of valuable natural products. They inhabit plant tissues without causing harm, and their ability to produce pharmacologically significant metabolites has sparked growing interest. Endophytic fungi have successfully synthesized key compounds like taxol, azadirachtin, vincristine, and quinine, showcasing their potential in agriculture, medicine, and environmental applications. While widely recognized for their role in producing novel drugs, the molecular mechanisms of plant–endophyte interactions remain underexplored. This article reviews trends in endophyte-mediated biosynthesis, key success stories, current challenges, and future directions in endophyte-based drug discovery [400]. The synthesis of target derivative metabolites by fungal endophytes through biotransformation also shows promise in the research area of drug discovery. Ongoing research into the molecular mechanisms of plant-endophyte interactions holds promise for the innovation of unique compounds and sustainable improvement of medicinal plants, which might positively affect both human health and the environment.

## Data Availability

The datasets generated during and/or analyzed during the current study are available from the corresponding author on reasonable request.
